# Polymer Stabilized Cholesteric Liquid Crystal Siloxane for Temperature-Responsive Photonic Coatings

**DOI:** 10.3390/ijms21051803

**Published:** 2020-03-06

**Authors:** Weixin Zhang, Johan Lub, Albertus P.H.J. Schenning, Guofu Zhou, Laurens T. de Haan

**Affiliations:** 1 SCNU-TUE Joint Lab of Device Integrated Responsive Materials (DIRM), National Center for International Research on Green Optoelectronics, South China Normal University, Guangzhou 510006, China; W.Zhang1@tue.nl (W.Z.); a.p.h.j.schenning@tue.nl (A.P.H.J.S.); 2 Guangdong Provincial Key Laboratory of Optical Information Materials and Technology & Institute of Electronic Paper Displays, South China Academy of Advanced Optoelectronics, South China Normal University, Guangzhou 510006, China; 3 Laboratory of Stimuli-Responsive Functional Materials & Devices, Department of Chemical Engineering and Chemistry, Eindhoven University of Technology, P.O. Box 513, 5600 MB Eindhoven, The Netherlands; J.Lub@tue.nl; 4 Academy of Shenzhen Guohua Optoelectronics, Shenzhen 518110, China

**Keywords:** cholesteric liquid crystal elastomers, polymer coatings, stimuli-responsive materials

## Abstract

Temperature-responsive photonic coatings are appealing for a variety of applications, including smart windows. However, the fabrication of such reflective polymer coatings remains a challenge. In this work, we report the development of a temperature-responsive, infrared-reflective coating consisting of a polymer-stabilized cholesteric liquid crystal siloxane, applied by a simple bar coating method. First, a side-chain liquid crystal oligosiloxane containing acrylate, chiral and mesogenic moieties was successfully synthesized via multiple steps, including preparing precursors, hydrosilylation, deprotection, and esterification reactions. Products of all the steps were fully characterized revealing a chain extension during the deprotection step. Subsequently, the photonic coating was fabricated by bar-coating the cholesteric liquid crystal oligomer on glass, using a mediator liquid crystalline molecule. After the UV-curing and removal of the mediator, a transparent IR reflective polymer-stabilized cholesteric liquid crystal coating was obtained. Notably, this fully cured, partially crosslinked transparent polymer coating retained temperature responsiveness due to the presence of non-reactive liquid-crystal oligosiloxanes. Upon increasing the temperature from room temperature, the polymer-stabilized cholesteric liquid crystal coating showed a continuous blue-shift of the reflection band from 1400 nm to 800 nm, and the shift was fully reversible.

## 1. Introduction

Stimuli-responsive photonic polymer coatings are able to change color in response to an external stimulus [1]. These “smart” coatings have potential applications in energy-regulating windows [2,3], food safety labels, healthcare products and smart packaging [1]. Many photonic systems have been developed using cholesteric liquid crystals (Ch-LCs) [4]. Ch-LCs are molecular assemblies with a helical arrangement. When well aligned, they act as Bragg reflectors that selectively reflect light of a specific wavelength while being transparent to others. The reflection wavelength of a Ch-LC is dependent on its helical pitch and can be tuned by the concentration of a chiral dopant present in the Ch-LC mixture [5]. The temperature-responsive shift of the Ch-LC reflection band has been realized in glass cells by changing the helical pitch. This change can be achieved by either using temperature-sensitive chiral dopants [6,7,8] or by utilizing the pre-transitional effect, which causes a strong redshift of the reflection band near the phase transition between the Ch-LC phase and the smectic (Sm) phase as a result of unwinding of the helix [9,10,11,12]. To obtain stimuli-responsive photonic coatings which would enable the industrial production of foils, polymers are desired [13]. Complete crosslinked LC polymers fail to retain any pre-transitional effect from their precursors [14,15]. A polymer-stabilized liquid crystal (PSLC) in a cell containing 5 wt% of crosslinked network manages to partially display the pre-transitional effect [16]. However, this PSLC system contains mostly low-molar-weight LCs which are unstable as coatings, because dewetting and slow evaporation may occur.

Polymeric materials are required for stable-performance photonic coatings. Photonic coatings of temperature-responsive non-polymerized Ch-LC mixtures encapsulated in polymer protective layers were recently reported, showing a good stability and bandshift range by the Sm-Ch pre-transitional effect, but a subtle stratification process is required for their fabrication [17,18,19]. Alternative ways to make temperature-responsive photonic polymer coatings use either the humidity [20] or heat expansion of the materials [21,22], which are not able to induce a large reflection band shift without the presence of water. Soft polymers such as polysiloxane Ch-LC elastomers or semi-interpenetrating polymer networks based on oligosiloxanes or a Ch-LC polymer network have been reported that showed temperature-responsive optical changes [23,24], but the shift of the reflection band was limited. Additionally, the cholesterol group was selected as a chiral generator in these polymers, leading to their responding temperatures being much higher than room temperature (>100 °C). Therefore, the fabrication of a photonic polymer coating with large temperature responsiveness under moderate temperatures remains a challenge.

**Scheme 1:**
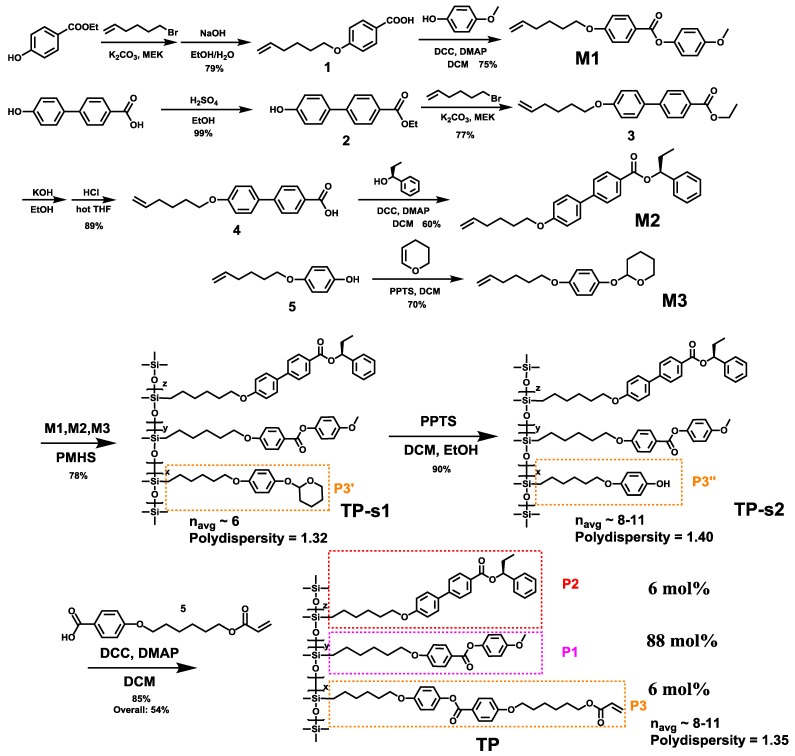
Synthetic routes for the preparation of the terpolymer (TP) and the required precursors M1, M2, and M3.

In our previous work, we prepared a temperature-responsive coating by blade-coating a mixture of oligomeric polysiloxane and a chiral dopant [25]. This non-crosslinked Ch-LC coating possessed a Sm-Ch phase transition, leading to a ~1200 nm bandshift within the IR region upon changing the temperature between room temperature and 66 °C. In this work, we developed a photopolymerizable, Ch-LC polysiloxane mixture for the preparation of a temperature-responsive polymer network-stabilized photonic coating. We designed and prepared a siloxane terpolymer (TP) with reactive acrylate mesogenic side groups (Scheme 1). The synthesis of the TP was performed via a three-step route: hydrosilylation, deprotection, and esterification. From the molecular weight of the final polydispersed terpolymer, an average estimated chain length of approximately 8~11 was determined. This TP was processable into a planarly aligned Ch phase coatings by bar-coating, with the help of a nematic solvent that mediates the alignment. With the full conversion of the acrylate groups under UV-initiated polymerization, crosslinked networks were obtained in the coating. This partially crosslinked polymer coating still shows a ~600 nm reflection band shift within the IR region in response to a temperature change between 20–66 °C, owing to the Sm-Ch phase transition. Such temperature-responsive polymer-stabilized polysiloxane photonic coatings would be attractive for a variety of applications including foils for energy-saving windows [3,26,27,28,29,30,31].

## 2. Results

### 2.1. Synthesis and Characterization of the CLC Oligosiloxane Mixture

We designed a side-chain polysiloxane LC terpolymer with both cholesteric and smectic LC phases and that is crosslinkable (TP, Scheme 1). This polysiloxane-derived terpolymer contained three mesogenic side groups (P1, P2, and P3) prepared from three precursor molecules (M1, M2, and M3), which were attached to the polysiloxane backbone by hydrosilylation. Mesogenic side group P1 induces a Sm-N transition in polysiloxane polymer [32,33], the chiral side group P2 induces a Ch LC phase, and the acrylate-containing mesogenic group P3 enables photo-polymerization. Monomers M1, M2 and M3 were synthesized and fully characterized by ^1^H NMR and ^13^C NMR (Figures S1–S5). Although the complete synthesis of M1 has been reported [25,34], here, we use a new approach to synthesize the intermediate product, 4-(hex-5-en-1-yloxy)benzoic acid (1) with a higher yield, by using ethyl 4-hydroxybenzoate as the reactant followed by dehydration of the ester group. The chiral dopant M2 is a new molecule designed to achieve a prominent helical twisting effect at low concentration. Synthesis of M2 was performed in four steps, with an overall yield of 40.7%. The helical twisting power (HTP) of M2 was estimated to be ~21 μm^−1^ in E7 (by wt%). M3 contains a protecting group which was used to prepare the acrylate-containing mesogenic group after the hydrosilylation reaction. It was used instead of the final mesogenic group to avoid participation of the acrylate group during the hydrosilylation.

The TP was obtained from the precursors and oligo(methylhydroxylsiloxane) (OMHS) in three steps of synthesis (Scheme 1). M1 represented the vast majority of precursor used (88 mol%), while M2 represented 6 mol% of the amount of precursor to obtain a cholesteric phase with a reflection band in the IR region. The amount of precursor M3 was 6 mol% in order to induce a low crosslinked ratio after photo-polymerization and to maintain a proper pre-transitional effect. The OMHS used had an average chain length (n_avg_) of 3.9. The polymer backbone was determined to be short in order to obtain only a small mol% of crosslink-able oligomers, which also maintained a low crosslinked ratio in the coatings. In the first synthesis step, TP-s1 was made through a hydrosilylation reaction of M1, M2, and M3 with PMHS, in a yield of 77.6%. The product TP-s1 was found to have a n_avg_ of approximately 6, calculated based on the ^1^H NMR spectrum (Figure S6; the formulation can be found in [25]). The increase of n_avg_ is most likely due to the loss of the low-mass fraction during precipitation. In the second step, the tetrahydropyran (THP)-protecting group was removed in the presence of pyridinium p-toluenesulphonate and ethanol to form TP-s2. This deprotection reaction that formed the free phenolic group was completed without affecting the other mesogen side groups (see in ^1^H NMR in Figure S7). However, gel penetration chromatography (GPC) of TP-s2 (Figure S10) revealed that the average molecular weight (M_W_) of TP-s2 had increased by 42% compared to TP-s1. Furthermore, a wider molecular weight distribution (polydispersity increased from 1.33 to 1.40) was observed. This molecular weight increase was disproportional to the weight loss of this step (yield = 90%). Moreover, a considerable number of cyclic oligomers was found in the matrix-assisted laser desorption/ionization-time of flight mass spectrometry (MALDI-TOF-MS) spectrum, which was absent in TP-s1 (Figure S11). Among the signals of the cyclic oligomers, the most intense ones are found at 1568, 1955, and 2342 Da and can be attributed to the four-ring, five-ring, and six-ring homo-oligomer of P1 as M^-^Na^+^ adducts, respectively. Trace signals of cyclic co-oligomers with one and two P2 or P3′’ units are present in the spectrum as well. The Si-OH group or trifunctional branching silicon atoms could not be detected using the ^29^Si NMR of TP-s2 (Figure S7). Based on these results and literature reports on similar phenomena [35], we assume that an exchange of the Si-O-Si bonds among the siloxane backbones was catalyzed by the mildly acidic PPTS, resulting in the appearance of both longer chains and cyclic compounds.

**Figure 1:**
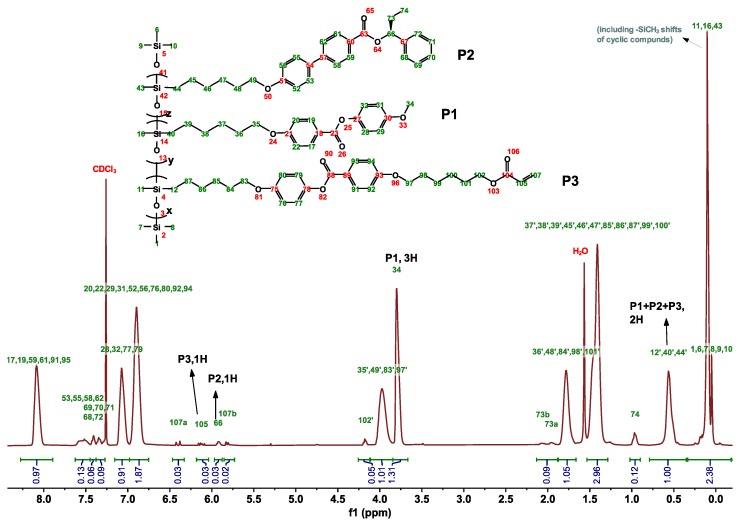
^1^H NMR spectrum of TP, with peak assignments and peak integral values.

**Figure 2:**
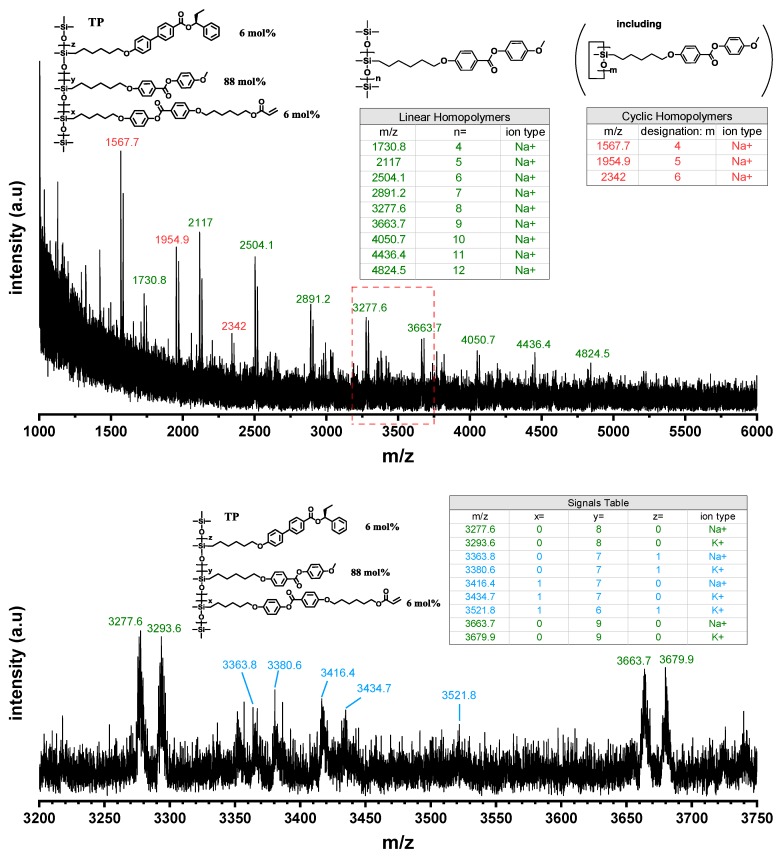
Matrix-assisted laser desorption/ionization-time of flight mass spectrometry (MALDI-TOF-MS) spectrum (top) and zoom in (bottom) of TP, with captions indicating different species.

The third reaction step, the esterification of the phenol groups in TP-s2 with carboxylic acid building block 5 using N,N′-dicyclohexylcarbodiimide (DCC) and N,N-4-dimethylaminopyridine (N,N-4-dimethylaminopyridine), yielded the desired terpolymer (TP). The ^1^H NMR spectrum of TP (Figure 1) confirmed the correct chemical structure of the polymer. This data also confirmed the molar ratio of the Sm-N mesogen (P1), chiral dopant (P2), and acrylate mesogen (P3) units to be 88:6:6. The molecular weight of TP was found to be 5012 Da, with a polydispersity index of 1.36, by GPC (Figure S10). MALDI-TOF-MS spectra (Figure 2) confirmed the molecular weight of the differently distributed mesogenic species of the polymer. Cyclic chain oligomers generated from TP-s2 remained in the final product but were considered a minor fraction (see also ^29^Si NMR spectrum of TP, Figure S8). Based on these results, we estimated a n_avg_ of TP between 8 and 11. From this estimated n_avg_ and the total concentration of crosslinker in the oligomers, it can be seen that a considerable fraction of the oligomer molecules in the TP do not contain any acrylate group (50.6–60.1%, see the calculations in Table S1), and, therefore, these molecules will definitely not participate in the network formation. Creating a crosslinked network requires molecules that contain at least two acrylate groups. Through these calculations, we also find that the chance of TP oligomers having >2 acrylate groups is not trivial (7.9 mol% when n_avg_ = 8). Therefore, a rather high network fraction after crosslinking is expected.

**Figure 3:**
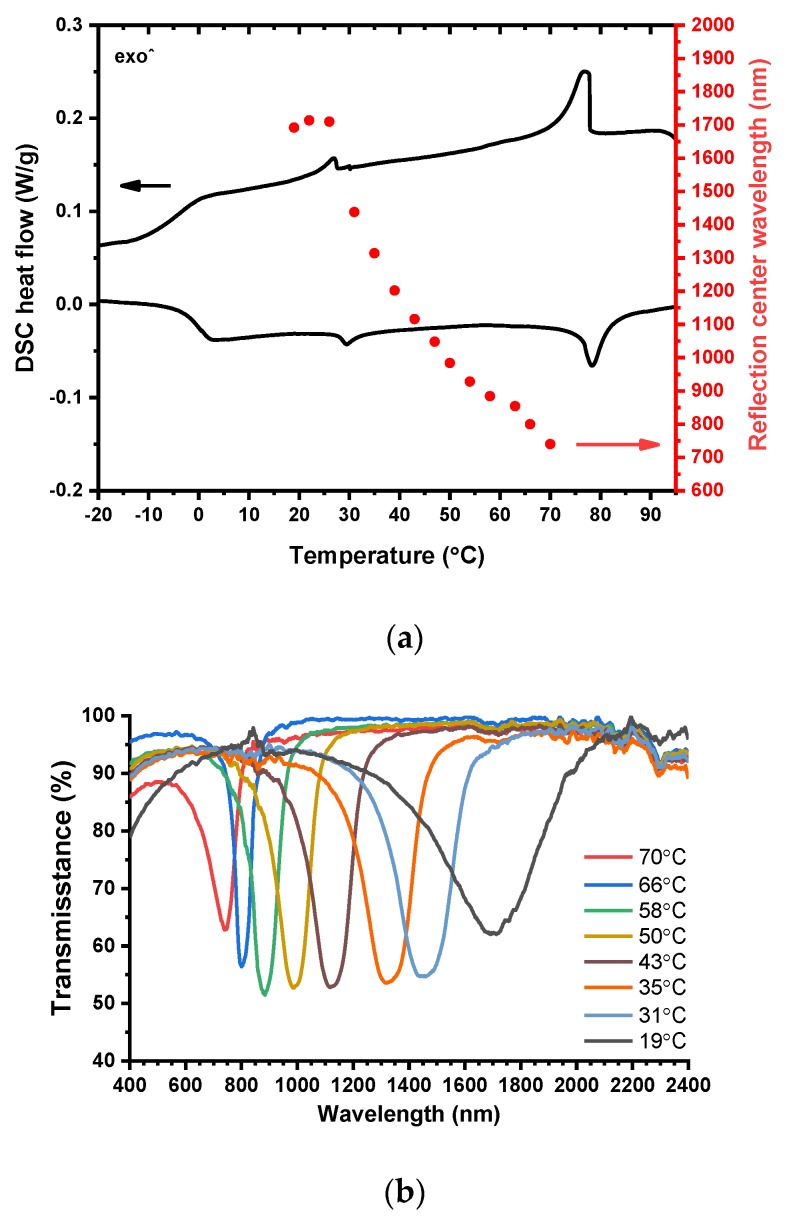
(**a**) Different scanning calorimetry (DSC) curves of TP (black lines), and center of the reflection band as a function of temperature (red dots). (**b**) Transmission spectra of the TP when filled in an alignment cell.

The liquid crystalline properties of the TP were subsequently examined using differential scanning calorimetry (DSC). Despite being a complex mixture of various species, TP shows clear peaks of phase transitions on DSC curves (Figure 3a). Isotropic transition and Sm-Ch transition were observed at 78 °C and at 29.5 °C, respectively. The glass transition (Tg) of the material was around 0 °C. The TP had a clear cholesteric reflection band below 70 °C, which was measured by the transmission spectroscopy of a TP-filled cell with planar alignment layers. Figure 3a,b shows that the reflection wavelength continuously increased as the temperature decreased and stabilized below the transition point of 29.5 °C. This confirmed a Sm-Ch phase pre-transitional effect of this liquid crystalline polymer.

### 2.2. Fabrication of Polymer Stabilized Cholesteric Liquid Crystal Siloxane Coating

**Scheme 2:**
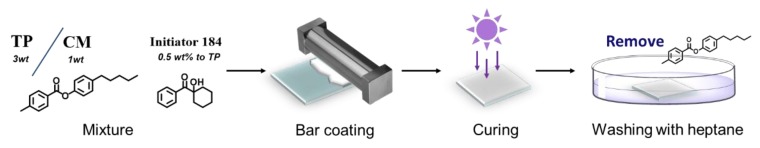
Schematic of the preparation and UV curing of terpolymer coatings.

We coated the TP on a glass substrate with an easy-to-conduct bar-coating method. The fabrication of the coatings is shown in Scheme 2. The mixture was first bar-coated on glass prepared with a rubbed polyimide layer to obtain a planar Ch alignment. This was preferably performed right below the isotropic transition, where the material had a lower viscosity, in order to achieve planar cholesteric alignment under shear force [36]. However, coating the pure TP did not result in a satisfying planar Ch alignment, as the viscosity was too high, causing all mesogens to be aligned uniaxially along the coating direction (Figure S12). To lower the viscosity and improve the alignment of the material, we added a coating mediator CM to the terpolymer. CM is an apolar nematogenic molecule [37] with structural similarity to the majority mesogenic side group P1 (Scheme 2) of TP, which ensured good mixing without disrupting the LC phase. CM was lab-synthesized via anesterification step (see Supplementary Information) and its structure was confirmed by ^1^H NMR spectrum (Figure S9). Indeed, a mixture of CM with TP in a weight ratio of 1:3 was still liquid crystalline. This 1:3 mixture had the right properties to coat and align the material at the cholesteric temperature of 51 °C. In addition, 0.5 wt% of photoinitiator Irgacure 184 was added to the mixture to initiate photo-polymerization of the acrylate groups in TP. This concentration was more than five times larger compared to the commonly used initiator concentration in reactive mesogen (RM) systems [38,39] to ensure the full conversion of acrylate groups in TP (Figure S13).

**Figure 4:**
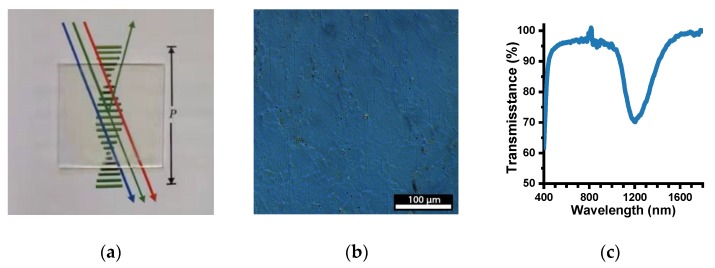
(**a**) Image of a fully-cured TP coating at room temperature, with a colorful image as background showing good visible light transparency. (**b**) Polarizer microscopy (POM) image of a fully-cured TP coating at room temperature (transmission mode), showing a planar aligned cholesteric texture. The bluish color might be due to the reduced efficiency of the polarizers. (**c**) Transmission spectrum of the fully cured coating at room temperature, after the removal of CM.

The coating was irradiated with UV under nitrogen, during which acrylate groups were polymerized. Finally, after cooling, the coating was washed in heptane multiple times to remove the CM molecules from the coating (see Section 4). The fabricated pristine coatings showed good visible light transparency at room temperature (Figure 4a). Polarized microscope images of the coating presented a planarly aligned Ch texture (Figure 4b), and a reflection band centered at ~1200 nm was seen in the transmission spectrum of the coating (Figure 4c). These results indicate that the fully cured coating retained a proper planar Ch alignment, even after the extraction of CM with heptane. The coating was still partially soluble in dichloromethane. After suspension in dichloromethane and filtration, the dried residue comprised ~4 wt% of the original coating. GPC analysis of the filtrate (Figure S21) showed that it contained large macromolecules consisting of multiple linked TP molecules which were not part of the continuous network (see the Supplementary Information for more information).

### 2.3. Temperature Response of the Polymer Stabilized Ch-LC Siloxane Coating

**Figure 5:**
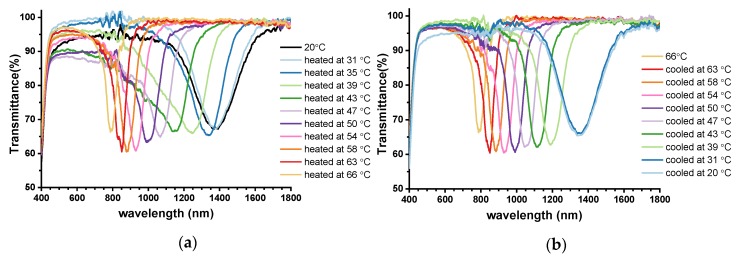
Transmission spectra of the fully cured TP coating recorded during the second heating round (**a**) and the second cooling round (**b**). The thickness of the tested coating was 12 μm.

The thermochromic properties of the polymer-stabilized Ch-LC coatings were investigated using UV-Vis-NIR spectroscopy. The absorption spectra of the coatings were measured at different temperatures during stepwise heating and cooling cycles. The first cycle of heating and cooling was different from the rest of the cycles. It is most likely that the Ch helix reassembles from the collapse caused by the removal of CM during the fabrication of the coating. Further heating and cooling give the same temperature-responsive optical changes, and multiple cycles of heating and cooling show repetitive results (Figure S14). Figure 5 shows the spectra of the coating during the second heating and cooling cycle. Increasing the temperature stepwise from 31 °C to 66 °C resulted in a ~600 nm blueshift of the reflection band center, from 1363 nm to 775 nm (Figure 5a). Using p = λ/n_avg_, with p the pitch length and n_avg_ the average refractive index, the corresponding pitch length is estimated to change from 885 nm to 503 nm, assuming the average refractive index = 1.54 [40]. A reversed redshift was seen during cooling steps (Figure 5b). Below 31 °C, the reflection band redshift ceased. The reflection band was stable at room temperature (20 °C) for at least 48 h (Figure S15). The fully-polymerized coating maintained a similar responsiveness to the uncured coating and to the original polymer in an alignment cell at 31~66 °C, as seen in Figure S16. Upon heating above 66 °C, the reflection band vanished due to isotropization, and upon cooling to Ch, the temperature did not reverse to the transparent-reflective state, but to an intensely light-scattering state, indicating that the planar Ch structure was destroyed (Figure S17). The kinetic behavior of the TP coating was studied from these spectra (Figures S18 and 19), showing a typical Sm-Ch pre-transitional kinetic effect [25]. The DSC curve of the fully cured coating confirmed the Sm-Ch transition is still present in TP (Figure S20), at 29 °C, which is right around the temperature threshold that the reflection band ceases to shift.

## 3. Discussion

The temperature-dependent transmission data indicate that the Sm-Ch transition is still the driving force behind the reflection band shifting of the TP coating after crosslinking. It is interesting to see this well-preserved pre-transitional effect in this polymer-stabilized PSLC coating, as the effect is usually strongly suppressed and in most cases disappears completely after crosslinking has been applied. In a previously reported example of a PSLC system in a cell containing small LC molecules and a 5% crosslinked RM network, the pre-transitional effect of the Sm-Ch LCs was strongly reduced after the network was formed [16], and in crosslinked polymer networks with no small molecules present, this has never been observed. In the PSLC coating presented here, 31.1–35.5% of the TP has one acrylate side group and 7.9–13.9% has two, implying a higher crosslink density; however, it maintained most of the pre-transitional effect after crosslinking.

The investigations on the polymer crosslinking of the completely cured coating (see the Supplementary Information for more information) showed that it consists of unreacted TP (presumably without an acrylate side group), large macromolecules consisting of multiple TP molecules linked together, and a continuously crosslinked network. This is different from traditional PSLC systems which only contain small molecules and a continuous network [41,42]. We propose that the polymerization mechanism of the current system is very different to the traditional PSLC systems in which continuous polymer networks are observed [41,42], as the high viscosity of the material limits polymer chain propagation. As a result, most of the short networks are hindered from connecting with each other to form larger networks, and only a relatively small fraction of the TP becomes part of the continuous network. However, it seems that the responsiveness of the TP coatings benefits from the different way of crosslinking in this system, though some of the improvements can be attributed to the more flexible siloxane backbone compared to RMs.

## 4. Materials and Methods

### 4.1. Materials

Photoinitiator Irgacure 184 was purchased from Ciba Specialty Chemicals Inc (Basel, Switzerland). Other materials were lab-synthesized. For details of the syntheses, see the Supplementary Information.

### 4.2. Mixtures

The TP and CM were weighed in brown glass vials, in a weight ratio of 3:1. Subsequently, initiator 184 was added via solution in a volumetric flask (2 mg/mL in unstabilized THF), precisely in 0.25 wt% or 0.5 wt% to the weight of TP. More unstabilized THF was then added to dissolve all components, leading to a solution of ~45 wt% in the end.

### 4.3. Preparation of Rubbed Polyimide Coated Glass Substrates

The same procedure was followed as in [43].

### 4.4. Preparation of Coatings

An RK PrintCoat Instruments K control coater was used to prepare the coatings. The mixtures were applied on polyimide-rubbed glass substrates and placed at 60 °C for 1 h to evaporate the solvent. Then, the coatings were applied using a 30 µm gap (4-sided applicator, ZFR 2040.8030, Zehntner, Sissach, Switzerland), which was pushed forward over the mixture automatically by the coater. The temperature of the substrate surface during the application was approximately 51 °C. The speed of the applicator movement was 0.5 cm/s. The coatings were then cooled down to 43–47 °C at the surface, flushed with nitrogen constantly for 1.5 h, and illuminated with UVA using an EXFO Omnicure S2000 mercury lamp, at an intensity of 20 mW/cm^2^ for 10 min. After the exposure, the coatings were kept in nitrogen atmosphere for 1h, and were cooled down to room temperature. CM component was removed from the coatings completely, by dipping the coatings in heptane for at least 16 h, with an exchange of solvent three times. The coatings were then collected and dried in air.

The thickness of the obtained coatings was measured using a Fogale Zoomsurf 3D interferometer. The coatings fabricated for optical measurements were ~11–13 μm in thickness.

For the control experiment of the mixture without using initiator 184, the coating was cooled to room temperature right after the coating application without UVA curing.

For the samples used in the crosslink fraction tests, the mixtures were coated on 9 × 12 cm clean glass substrates without polyimide layers. A larger portion of materials was used to create larger coating samples.

### 4.5. Characterization

NMR spectra were recorded on a 400 MHz Bruker Avance III HD spectrometer. For ^29^Si measurements, Chromium(III) acetylacetonate and tetramethylsilane were added to the sample, and the measurements were performed under the inverse-grated mode. Matrix-assisted laser desorption/ionization time-of-flight mass spectrometry (MALDI-TOF MS) was performed on a Bruker Autoflex Speed MALDI-MS instrument, with a DCTB as matrix agent. Differential scanning calorimetry (DSC) curves were measured with a DSC Q2000, TA Instruments. Gel penetration chromatography (GPC) was performed on a Shimadzu LC-2030C.3D instrument equipped with a PDA-254 nm detector, using chloroform as the eluent and monodisperse polystyrene calibration standards. Polarizer microscopy (POM) images were taken on a Leica microscope DM2700 in transmission mode, equipped with polarizers.

### 4.6. Polymeric Distribution Analysis of the Coatings

The coatings were collected in 0.2 μm PTFE filters and weighed to find approximately 20 mg of materials on average. The filters containing these materials were flushed with a continuous flow of dichloromethane by syringes at room temperature, and then dried in a 40 °C oven. The flush-dry cycle was run until the weight of the dried filters did not reduce anymore. The crosslink fractions were calculated from the weight of the material left in the filter divided by the initial weight of material in the filter. The eluents were collected and analyzed with proton NMR and GPC.

### 4.7. Vis–NIR Transmission Spectra of the Coatings Over Temperature

Transmission spectra of the coatings were measured on a Shimadzu UV-3102 PC UV/Vis/NIR spectrophotometer equipped with a MPC-3100 sample compartment and a Linkam TMS93 temperature controller. A polyimide coated glass substrate was used as baseline. The actual temperature of the coating was calibrated using a thermometer probe (Comark KM340). A scan of spectrum started exactly every 5 min when the coating was kept at one temperature, until the new spectrum was identical to the previous one.

## 5. Conclusions

A polysiloxane Sm-Ch LC terpolymer with acrylate groups covalently linked to one of the mesogenic side-groups was successfully synthesized via a three-step route. The terpolymer obtained had an unexpectedly increased backbone chain length, and some formation of cyclic oligomer compounds was observed—both of which are side effects of the deprotection step. We propose the mechanism of these side reactions takes place through catalysis by PPTS. A temperature-responsive IR reflective coating was successfully fabricated from this terpolymer. This approach features easy processability using a bar-coating method. We have also found that a coating mediator can assist in the formation of planar alignment of cholesteric polysiloxane LCs during bar-coating. The fully-cured terpolymer coatings retained ~600 nm of reversible reflection band shift by heating and cooling. The partially retained temperature responsiveness of the coatings was still caused by the inherent Sm-Ch transitional effect of TP.

The approach of using a polymer-stabilized polysiloxane Sm-Ch type LC polymer to fabricate a temperature-responsive photonic polymer is promising. By adjusting the chiral component faction in the Ch-LC siloxane, a reflection color in the visible region could also be achieved, further broadening the range of potential applications. However, as far as applications are concerned, the synthesis and the use of a nematic solvent provide limiting factors. Future optimizations of the material should also focus on fine-tuning the response temperature range and enhancing the mechanical robustness of the coating.

## Supplementary Materials

Supplementary Materials can be found at https://www.mdpi.com/1422-0067/21/5/1803/s1.

## Abbreviations

Ch	Cholesteric
Sm	Smectic
LC	Liquid crystal
RM	Reactive mesogens
PSLC	Polymer-stabilized liquid crystals
