# Supplementary material for: Polymer Stabilized Cholesteric Liquid Crystal Siloxane for Temperature-Responsive Photonic Coatings
Source: Int J Mol Sci. 2020 Mar 6;21(5):1803. doi: 10.3390/ijms21051803 (PMC7084302; doi:10.3390/ijms21051803)
Supplement: Supplementary file 1 [file ijms-21-01803-s001.pdf]

Supplementary Information

Materials

Ethyl 4-hydroxybenzoate and 3,4-Dihydropyran were purchased from Shanghai Macklin Biochemical Co., Ltd. 6-bromohex-1-ene, 4'-hydroxy-[1,1'-biphenyl]-4-carboxylic acid, p-toluic acid and 4-pentylphenol were obtained from Beijing HWRK chemical Co., Ltd. 4-Dimethylaminopyridine DMAP, Dicyclohexylcarbodiimide (DCC), (S)-(-)-1-phenyl-1-propanol and 1-ethyl-(3-dimethylaminopropyl)carbonyldiimide hydrochloride (EDC•HCl) were obtained from Sigma-Aldrich. 4-(5-hexenyloxy)phenol (**5**) was synthesized by Philips Research Laboratories.[1] Pyridinium p-toluenesulfonate (PPTS) was purchased from Acros Organics. Poly(methyl hydrogen siloxane) (PMHS), trimethylsilyl terminated, average M_n~390, was purchased from Sigma-Aldrich (as the "OMHS"). n_{avg} of the received OMHS was found to be 3.88 by NMR. Platinum(0)-1,3-divinyl-1,1,3,3-tetramethyldisiloxane (Karstedt's catalyst), 2% in xylene solution, was purchased from Sigma-Aldrich. Anhydrous toluene was purchased from Acros Organics. 4-(6-acryloxy-hex-1-yloxy)benzoic acid was purchased from SYNTHON Chemicals GmbH & Co. KG.

Synthesis.

*Synthesis of 4-(hex-5-en-1-yloxy)benzoic acid (**1**)*

A solution was made of 50 g (0.3 mol) ethyl 4-hydroxybenzoate, 53 g (0.32 mol) 6-bromohex-1-ene and 52 g (0.376 mol) potassium carbonate in 750 mL butanone. The mixture was stirred at 85 °C overnight, then cooled to room temperature. After cooling, the solution was filtered and the solvent was evaporated. The residue was dissolved in 200 mL chloroform. The chloroform solution was extracted three times with an aqueous 1 N NaOH solution (3×200 mL) and with brine (2×200 mL). The organic phase was dried with magnesium sulfate, then a pale-yellow liquid was collected by solvent evaporation. This product was mixed with sodium hydroxide (25.2 g, 0.63 mol) ethanol (400 mL) and water (400 mL), and was refluxed for 12 hr. After being cooled to room temperature, the solution was acidified to a pH of 2 with concentrated HCl. The resulting precipitate was collected and recrystallized from ethanol (500 mL) as a white solid. Yield: 49 g, 79 %.

^{1}H NMR (500 MHz, Chloroform-d) δ 8.08 (d, J = 8.9 Hz), 6.95 (d, J = 8.9 Hz), 5.85 (ddt, J = 16.9, 10.2, 6.6 Hz), 5.04 (d, J = 2.2 Hz), 4.06 (t, J = 6.5 Hz), 2.20 – 2.13 (m), 1.95 – 1.76 (m), 1.73 – 1.51 (m).

*Synthesis of 4-methoxyphenyl 4-(hex-5-en-1-yloxy)benzoate (**M1**).*

To a solution of 8 g (36.3 mmol) 4-(hex-5-en-1-yloxy)benzoic acid (1), 4.66 g (37.6 mmol) 4-methoxyphenol and 0.42 g (3.44 mmol) DMAP in 220 ml DCM was added 8.73 g (42.3 mmol) DCC in 30 ml of DCM. The mixture was stirred overnight at room temperature. The suspension was filtered, and the solid was dried. The crude product was recrystallized three times from methanol, followed by column chromatography (pentane/DCM = 1:1). The final product was a white solid. Yield: 8.93 g, 75.4 %.

^{1}H NMR (400 MHz, Chloroform-d) δ 8.13 (d, J = 8.8 Hz, 2H), 7.12 (d, J = 9.0 Hz, 2H), 6.96 (d, J = 8.9 Hz, 2H), 6.93 (d, J = 9.0 Hz, 2H), 5.84 (ddt, J = 16.9, 10.2, 6.6 Hz, 1H), 5.05 (dd, J = 17.1, 1.7 Hz, 1H), 4.99 (dd,

J = 10.2, 1.5 Hz, 1H), 4.05 (t, J = 6.5 Hz, 2H), 3.82 (s, 3H), 2.20 – 2.09 (m, 2H), 1.90 – 1.78 (m, 2H), 1.65 – 1.54 (m, 2H).

*Ethyl 4′-hydroxy-[1,1′-biphenyl]-4-carboxylate (***2***)* was prepared according to literature methods.[2] Yield: 98.5 %.

*Synthesis of ethyl 4′-(hex-5-en-1-yloxy)-[1,1′-biphenyl]-4-carboxylate (***3***)*

8.00 g ethyl 4'-hydroxy-[1,1'-biphenyl]-4-carboxylate (**2**) (33 mmol), 6.00 g 6-bromo-1-ene (37 mmol), and 8.25 g K_2CO_3 (60 mmol) were dissolved in 180 mL butanone, followed by stirring at 85 °C for 42 h. After cooling, the solution was filtered, concentrated, dissolved in $CHCl_3$ (150 ml), washed with 1 N aqueous NaOH (~200mL), then washed with 1N aqueous NaCl (~200mL), then washed with water (~200mL), and dried with $MgSO_4$. After filtration, the solvent was removed under reduced pressure to give the crude product. The product was recrystallized from methanol to obtain the product as a white solid. Yield: 8.26 g, 77 %.

^{1}H NMR (400 MHz, Chloroform-d) δ 8.08 (d, J = 8.2 Hz, 2H), 7.61 (d, J = 8.2 Hz, 2H), 7.56 (d, J = 8.5 Hz, 2H), 6.98 (d, J = 8.7 Hz, 2H), 5.84 (ddt, J = 16.9, 10.2, 6.6 Hz, 1H), 5.05 (dd, J = 17.2, 1.7 Hz, 1H), 4.98 (dd, J = 10.2, 1.8 Hz, 1H), 4.39 (q, J = 7.1 Hz, 2H), 4.02 (t, J = 6.4 Hz, 2H), 2.20 – 2.09 (m, 2H), 1.89 – 1.77 (m, 2H), 1.66 – 1.53 (m, 2H), 1.41 (t, J = 7.1 Hz, 3H).

*Synthesis of 4′-(hex-5-en-1-yloxy)-[1,1′-biphenyl]-4-carboxylic acid (***4***)*

To a solution of 8.26 g ethyl 4'-(hex-5-en-1-yloxy)-[1,1'-biphenyl]-4-carboxylate (**3**) (25.5 mmol) in ethanol (100 mL) was added dropwise 9 g KOH (160 mmol) in ethanol (250 mL), followed by reflux for 40 h. After cooling, the precipitate was collected, rinsed with water, and then dissolved in hot THF and acidified with 2 N HCl. After cooling the precipitate was collected, washed with water, and recrystallized once from acetic acid. The resulting product was a white solid. Yield: 6.71 g, 88.9 %.

^{1}H NMR (400 MHz, DMSO-d_6) δ 12.94 (s, 1H), 7.98 (d, J = 8.3 Hz, 2H), 7.75 (d, J = 8.4 Hz, 2H), 7.68 (d, J = 8.7 Hz, 2H), 7.04 (d, J = 8.8 Hz, 2H), 5.84 (ddt, J = 16.9, 10.2, 6.6 Hz, 1H), 5.04 (dd, J = 17.1, 1.8 Hz, 1H), 4.98 (dd, J = 10.2, 1.9 Hz, 1H), 4.03 (t, J = 6.5 Hz, 2H), 2.16 – 2.05 (m, 2H), 1.80 – 1.68 (m, 2H), 1.59 – 1.46 (m, 2H).

^{13}C NMR (101 MHz, DMSO-d_6) δ 167.67, 159.44, 144.44, 139.02, 131.55, 130.41, 129.26, 128.58, 126.55, 115.46, 115.44, 67.84, 33.31, 28.59, 25.20.

*Synthesis of (S)-1-phenylpropyl 4′-(hex-5-en-1-yloxy)-[1,1′-biphenyl]-4-carboxylate (***M2***)*

0.72 g of (S)-(-)-1-phenyl-1-propanol (5.28 mmol), 0.063 g of DMAP (0.518 mmol) and 1.565 g of 4'-(hex-5-en-1-yloxy)-[1,1'-biphenyl]-4-carboxylic acid (**4**) (5.28mmol) was dissolved in 30 ml of dichloromethane. 1.088 g DCC (5.28 mmol) dissolved in 5 ml DCM was added dropwise to the mixture. The mixture was stirred at room temperature overnight. The resulting mixture was concentrated slightly, refrigerated, and then filtered. The eluent was collected, dried and further

purified by column chromatography (DCM/pentane 3 : 5). Recrystallizing 3 times from methanol gave the final product as a white solid. Yield: 1.31 g, 60 %.

^{1}H NMR (400 MHz, Chloroform-d) δ 8.19 (d, J = 8.4 Hz, 2H), 7.68 (d, J = 8.5 Hz, 2H), 7.61 (d, J = 8.8 Hz, 2H), 7.52 – 7.45 (m, 2H), 7.45 – 7.38 (m, 2H), 7.38 – 7.30 (m, 1H), 7.04 (d, J = 8.8 Hz, 2H), 6.00 (t, J = 6.8 Hz, 1H), 5.90 (ddt, J = 16.9, 10.2, 6.6 Hz, 1H), 5.11 (dd, J = 17.1, 1.8 Hz, 1H), 5.05 (dd, J = 10.3, 1.8 Hz, 1H), 4.06 (t, J = 6.4 Hz, 2H), 2.25 – 2.17 (m, 2H), 2.19 – 2.05 (m, 1H), 2.03 (ddq, J = 13.9, 7.4, 6.3 Hz, 1H), 1.95 – 1.82 (m, 2H), 1.71 – 1.58 (m, 2H), 1.04 (t, J = 7.4 Hz, 3H).

^{13}C NMR (101 MHz, Chloroform-d) δ 165.86, 159.38, 145.31, 140.73, 138.50, 132.28, 130.16, 128.58, 128.44, 128.34, 127.82, 126.49, 126.46, 114.93, 114.81, 77.82, 67.91, 33.45, 29.63, 28.71, 25.34, 10.01.

*Synthesis of **M3***

To a solution of 5 g of 4-(5-hexenyloxy)phenol (26 mmol) and 0.654 g PPTS (2.6 mmol) in 100 mL DCM was added 3.55 mL 3,4-dihydropyran (39 mmol), and the solution was stirred for 4 h at room temperature. Then the solution was concentrated, diluted with 200 mL diethyl ether, and washed with brine and water. Column chromatography over silica gel (pentane/DCM 5:3) resulted in a pale-yellow liquid product. Yield: 5 g, 69.6 %. Density: 1.047g/mL at 25°C.

^{1}H NMR (400 MHz, DMSO-d_6) δ 6.91 (d, J = 9.1 Hz, 2H), 6.80 (d, J = 9.1 Hz, 2H), 5.79 (ddt, J = 16.9, 10.2, 6.6 Hz, 1H), 5.27 (t, J = 3.5 Hz, 1H), 5.01 (dd, J = 17.2, 1.9 Hz, 1H), 4.94 (dd, J = 28.1, 17.2 Hz, 1H), 3.87 (t, J = 6.4 Hz, 2H), 3.81 – 3.70 (m, 1H), 3.54 – 3.43 (m, 1H), 2.17 – 1.99 (m, 2H), 1.92 – 1.34 (m, 10H).

^{13}C NMR (101 MHz, Chloroform-d) δ 154.16, 151.15, 138.72, 117.90, 115.36, 114.82, 97.48, 68.41, 62.23, 33.60, 30.65, 28.96, 25.49, 25.41, 19.09.

*Synthesis of **TP-s1***

Monomers **M1**, **M2**, and **M3** were put into a Schlenk flask in a molar ratio of 88 : 6 : 6, for a total of 11 mmol. 0.92 g OMHS was added to the flask, in which the amount of Si-H bonds was approximately 9 mmol. The components in the flask were flushed continuously with argon. 25 mL of anhydrous toluene was injected under argon atmosphere to dissolve the reactants. A catalytic amount of Karstedt's catalyst was then injected to start the reaction. The content of the flask was brought to reflux at 65 °C under argon atmosphere. The reaction was continued until sampling NMR showed no residual Si-H groups at δ=4.7. The finished reaction was cooled, and the product solution was precipitated in toluene/cold methanol (-20 °C) 3 times. After removal of the solvent in vacuum, the resulting solid was dissolved in toluene, and an adequate amount of silica gel metal scavengers (Si-Triamine, SilicaMetS, Silicycle Inc.) was added. This was followed by vigorous stirring overnight to remove the platinum catalyst from the product. The silica gel was removed through filtration and washed with toluene. The eluent was evaporated, yielding a white, viscous solid product. Yield: 3.01 g, 77.6 %. N_{avg} calculated from NMR using a literature method[3]: 6.12.

^{1}H NMR (400 MHz, $CDCl_3$) δ 8.10, 7.59, 7.52, 7.41, 7.35, 7.08, 6.90, 6.78, 5.93, 5.25, 3.99, 3.80, 3.56, 2.07, 1.96, 1.79, 1.65, 1.48, 1.41, 0.97, 0.55, 0.17, 0.10, 0.05. Peak assignments and integral values are given in Figure S6. Molar ratio of **P1**, **P2**, **P3'** units are confirmed as 88 : 6 : 6.

^{29}Si NMR (79 MHz, $CDCl_3$) δ 7.09, -22.29, -22.88, -22.99.

Synthesis of ***TP-s2***

3 g of **TP-s1** (containing ~0.47 mmol THP group) was dissolved in 40 ml DCM. 4 ml of ethanol (~100 mol. equiv. to THP amount) was added to the solution, 0.47 g of PPTS (0.188 mmol) was then added into the mixture with vigorous stirring. After 4.5 days the solution was precipitated into DCM/cold methanol three times. The product was a white and viscous solid. Yield: 2.7 g, ~90 %.

^{1}H NMR (400 MHz, $CDCl_3$) δ 8.10, 7.58, 7.52, 7.41, 7.35, 7.26, 7.08, 6.90, 6.70, 5.92, 3.97, 3.81, 2.06, 1.96, 1.78, 1.47, 1.41, 0.97, 0.56, 0.10, 0.05. Peak assignments and integral values are given in Figure S7. Molar ratio of **P1**, **P2**, **P3''** units are confirmed as 88 : 6 : 6.

^{29}Si NMR (79 MHz, $CDCl_3$) δ 7.09, -19.96, -22.29, -22.87, -22.99.

Synthesis of ***TP***

2.51 g **TP-s2** (containing ~0.4 mmol phenol group) was dissolved in 30 ml DCM. 0.585 g of 4-(6-acryloxy-hex-1-yloxy)benzoic acid (2 mmol, ~5 equiv.) and 0.0244 g DMAP (0.2 mmol) were added to the mixture. 0.412 g DCC (2 mmol, ~5 equiv.) was dissolved in 10 ml solution and added dropwise to the mixture. The mixture was stirred vigorously overnight. The solution was then concentrated and precipitated 3 times from DCM/cold methanol and once from toluene/heptane. Yield: 2.04 g, 85 %.

^{1}H NMR (400 MHz, $CDCl_3$) δ 8.07, 7.58, 7.41, 7.35, 7.07, 6.90, 4.16, 3.97, 3.80, 2.06, 1.96, 1.78, 1.46, 1.41, 0.97, 0.56, 0.19, 0.10, 0.05. Peak assignments and integral values are given in Figure 1.

^{29}Si NMR (79 MHz, $CDCl_3$) δ 7.11, -20.09, -22.28, -22.47, -22.86, -22.98.

Further analysis is shown in Session 2.1.

Synthesis of ***CM***

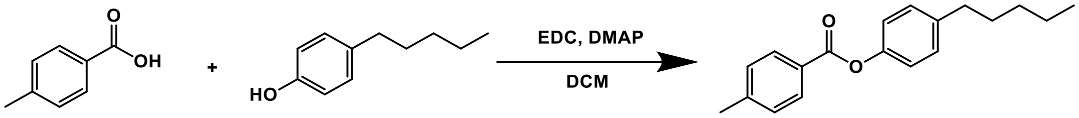

A solution of 1.36 g p-toluic acid (10 mmol), 1.64 g 4-pentylphenol (10 mmol), and 122 mg DMAP (1 mmol) in 100 mL DCM was cooled to 0 °C under argon, and 1.92 g (10 mmol) of EDC·HCl was added. After stirring overnight at RT, the solvent was removed under reduced pressure until 75 mL was left, and extracted subsequently with 50 mL of a saturated $NaHCO_3$ solution, 50 mL of water, and 50 mL of brine. After drying over anhydrous $MgSO_4$, the solvent was removed under reduced pressure. The crude product was then purified by column chromatography (hexane/DCM 7 : 1) and obtained as a white solid. Yield: 2.51 g, 89 %.

^{1}H NMR (600 MHz, Chloroform-d) δ 8.09 (d, J = 7.9 Hz, 2H), 7.31 (d, J = 8.0 Hz, 2H), 7.22 (d, J = 8.2 Hz, 2H), 7.11 (d, J = 8.7 Hz, 2H), 2.62 (t, J = 7.6 Hz, 2H), 2.45 (s, 3H), 1.67 – 1.59 (m, 2H), 1.40 – 1.29 (m, 4H), 0.91 (t, J = 6.9 Hz, 3H).

^{1}H NMR (400 MHz, $CDCl_3$) δ 8.10, 7.58, 7.52, 7.41, 7.35, 7.08, 6.90, 5.93, 3.99, 3.81, 2.08, 1.96, 1.79, 1.47, 1.41, 0.97, 0.55, 0.10, 0.05.

Characterization of the compounds.

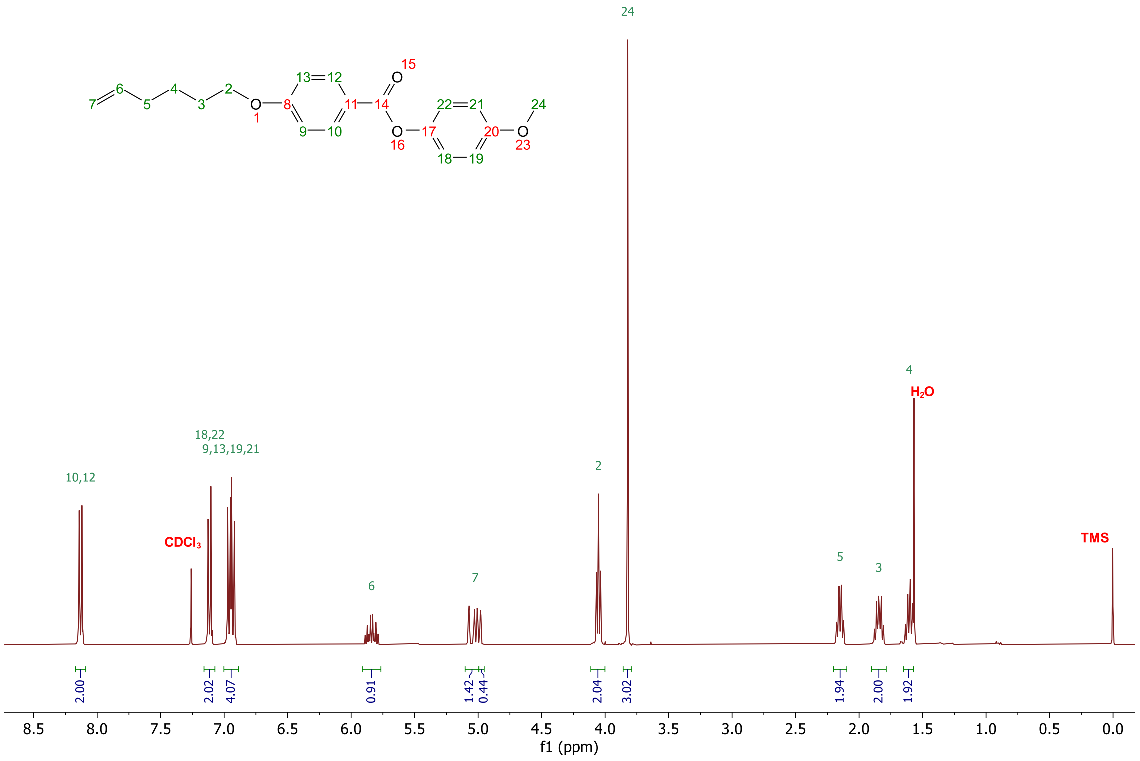

Figure S1. ^{1}H NMR spectrum of compound **M1**

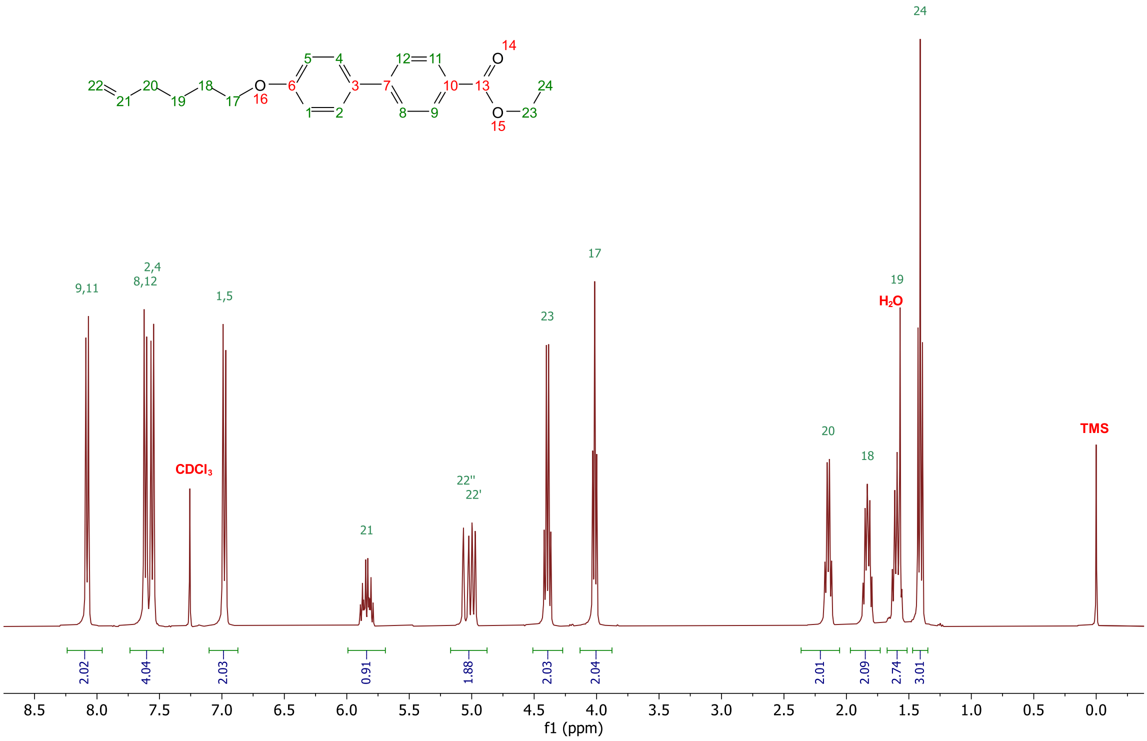

Figure S2. ^{1}H NMR spectrum of compound **3**.

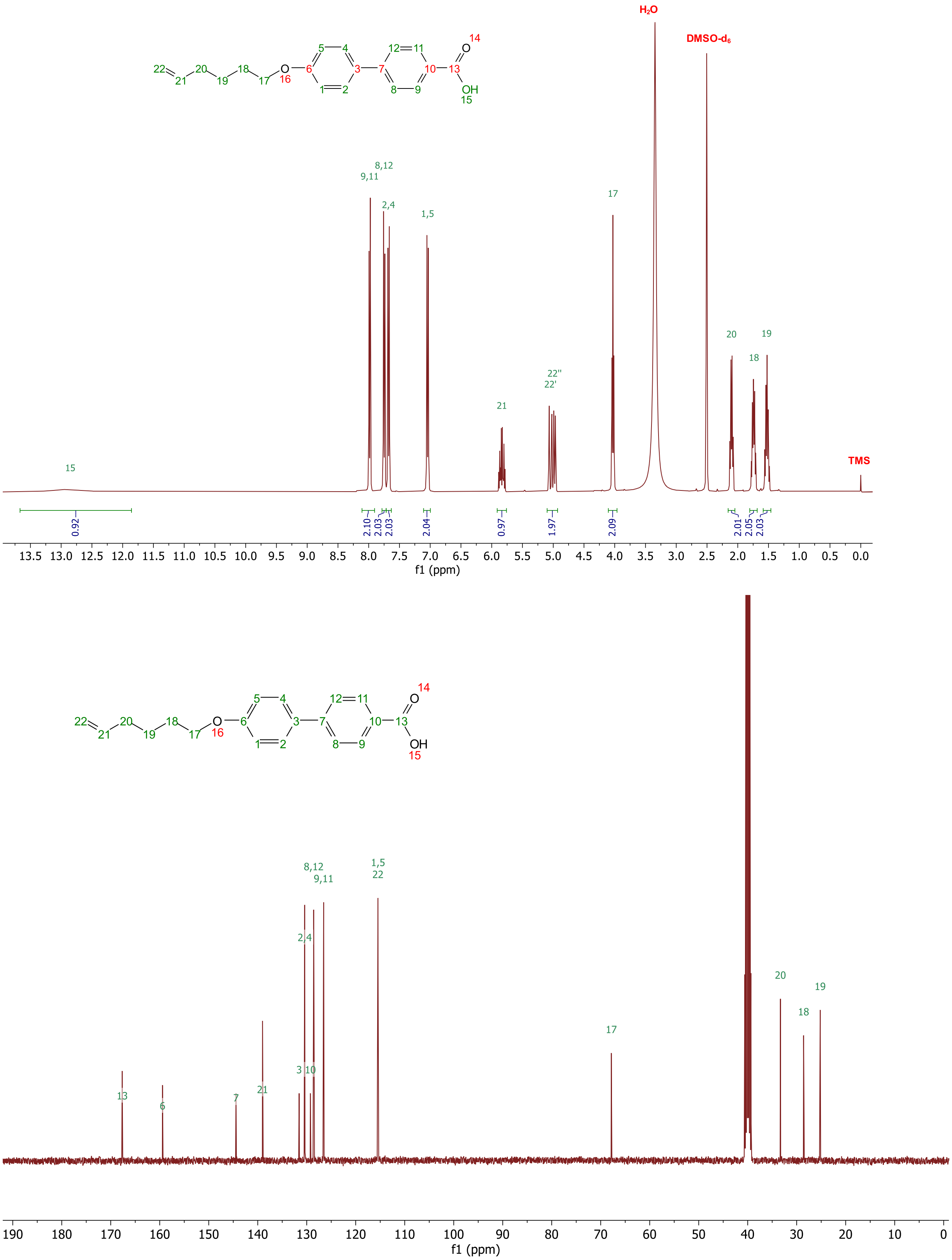

Figure S3. ^{1}H and ^{13}C NMR spectrum of compound **4**.

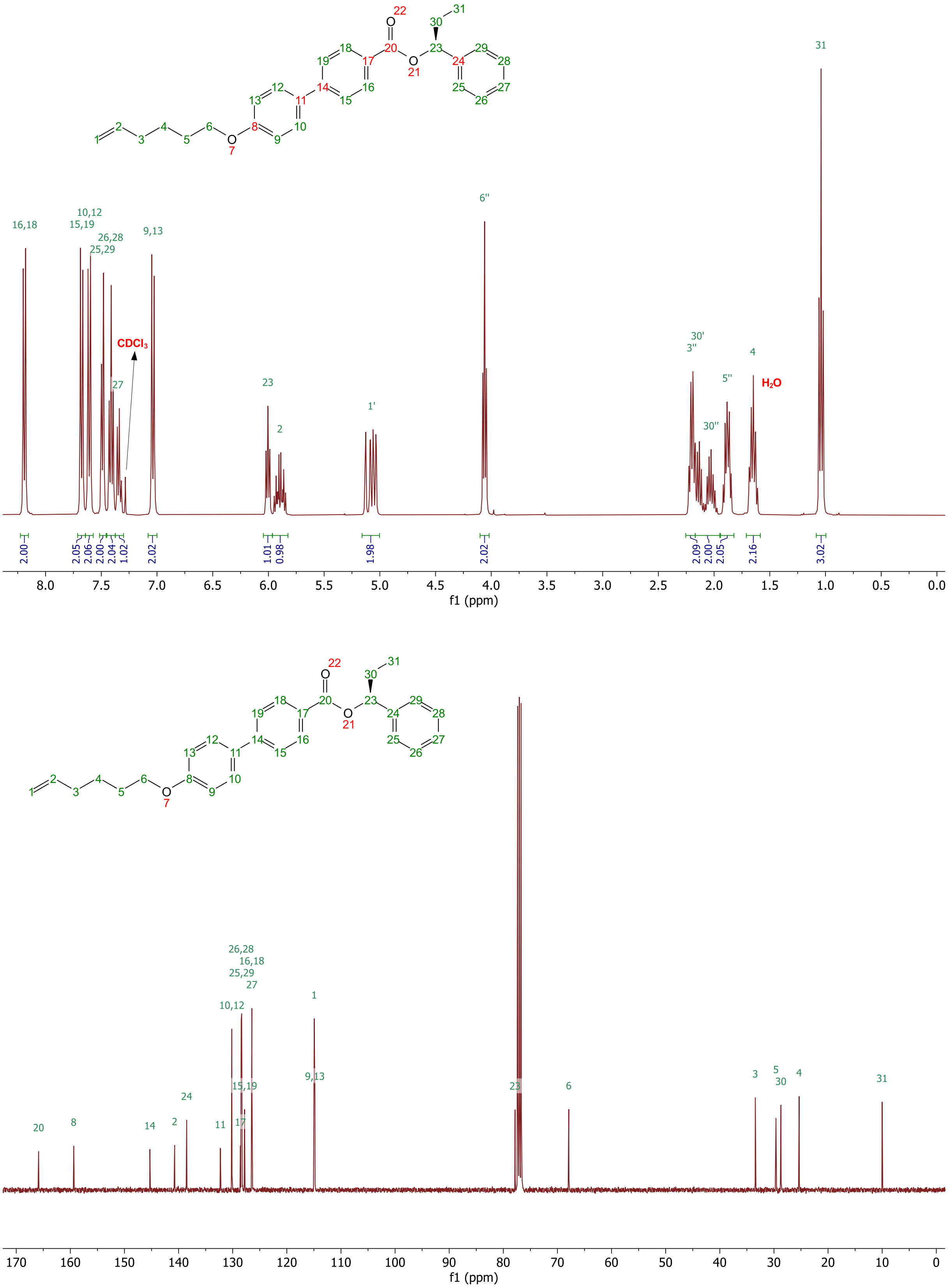

Figure S4. ^{1}H and ^{13}C NMR spectrum of **M2**.

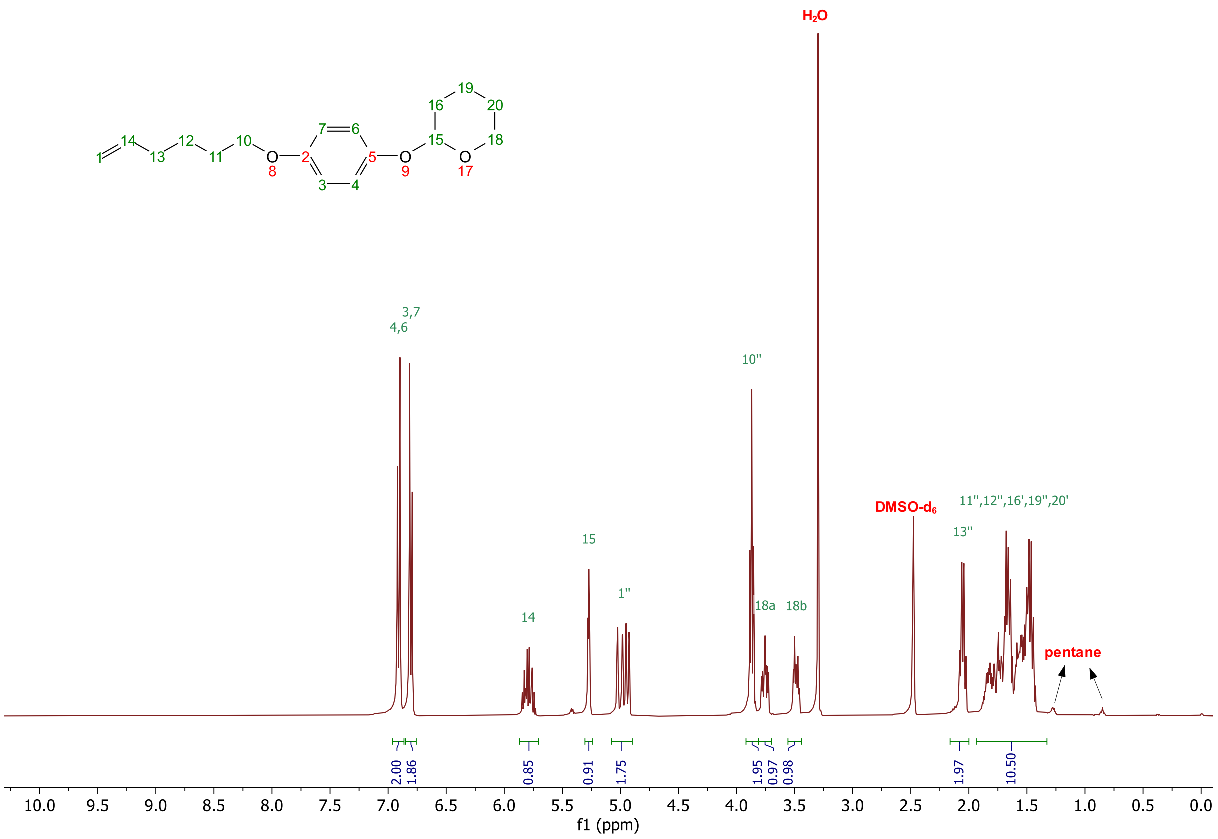

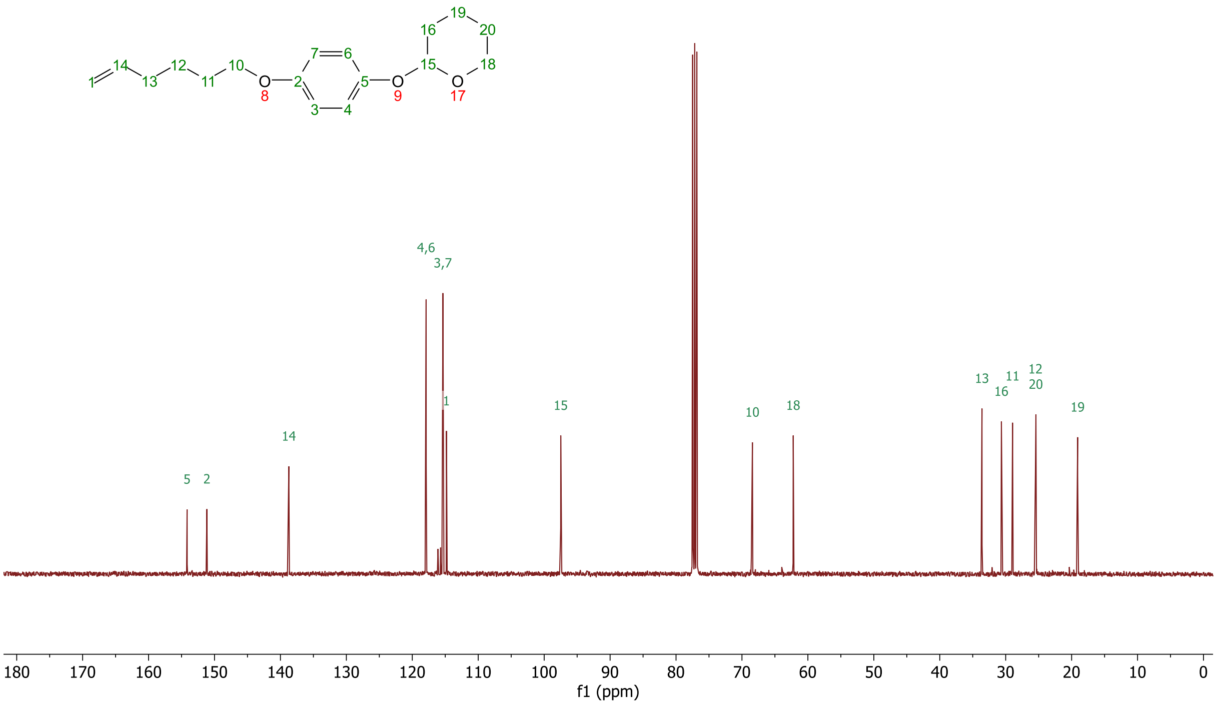

Figure S5. ¹H and ¹³C NMR spectra of **M3**.

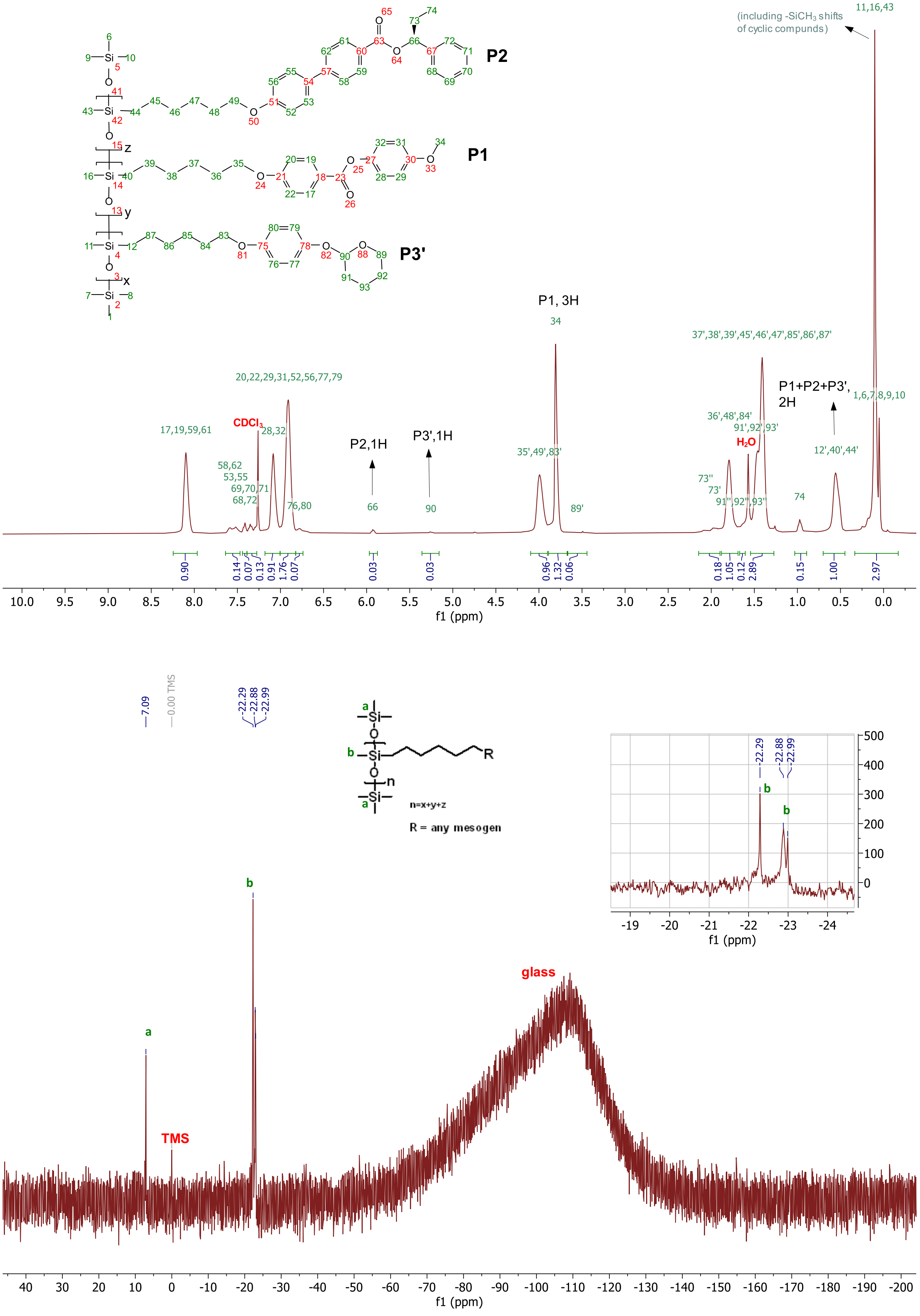

Figure S6. ^{1}H and ^{29}Si NMR spectrum of **TP-s1**. Assignment of the ^{29}Si NMR signals is based on literature references.[4,5]

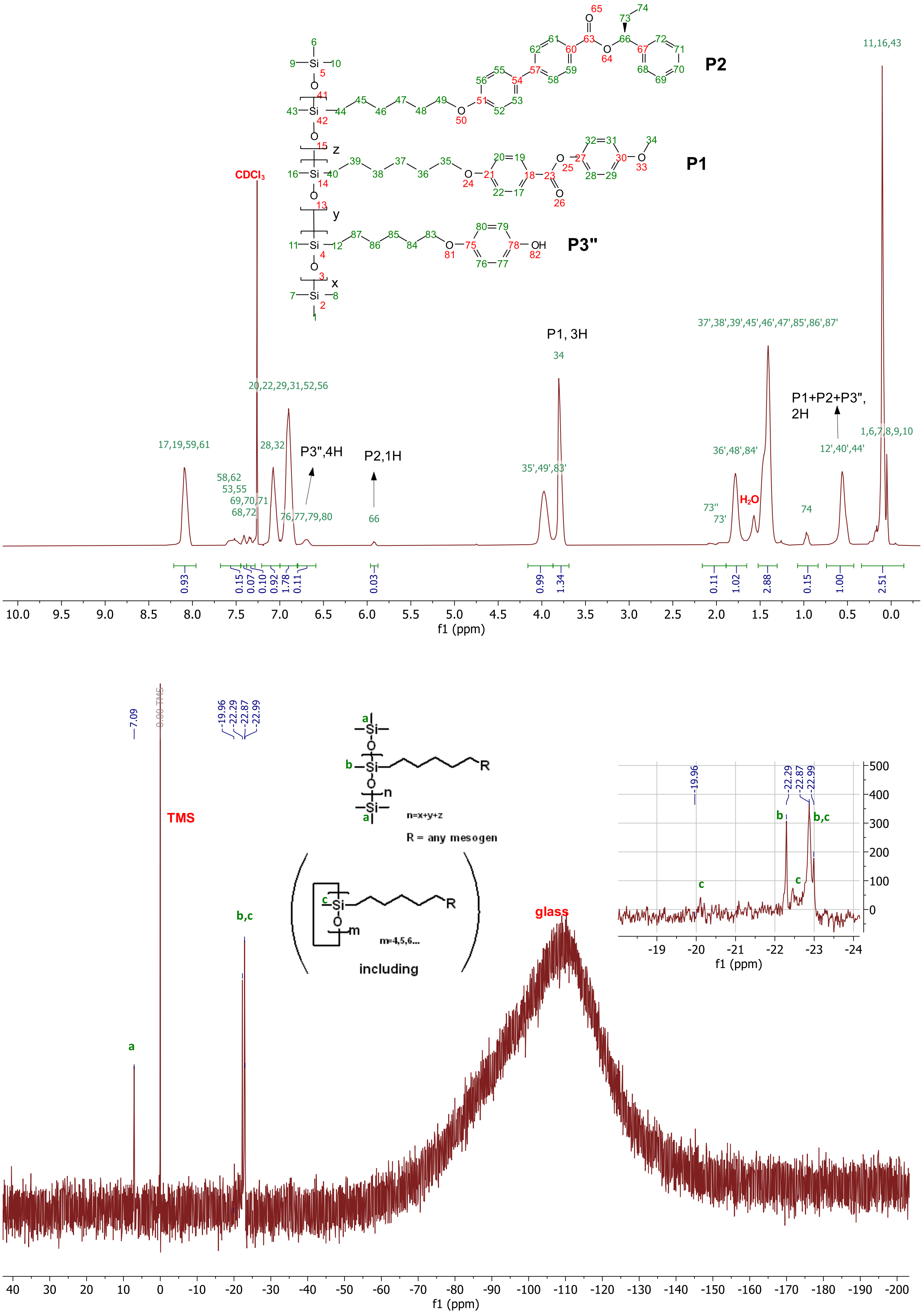

Figure S7. ^{1}H NMR and ^{29}Si NMR spectrum of **TP-s2**.

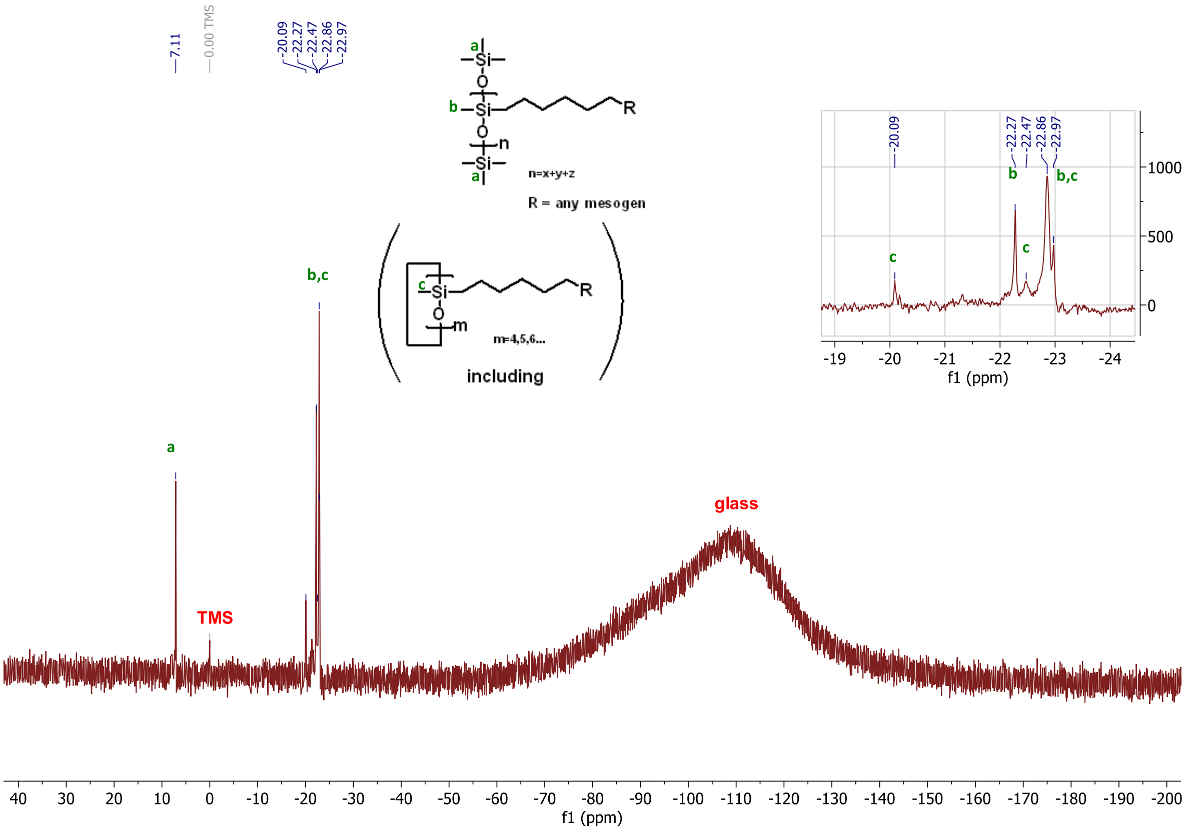

Figure S8. ^{29}Si NMR spectrum of **TP**

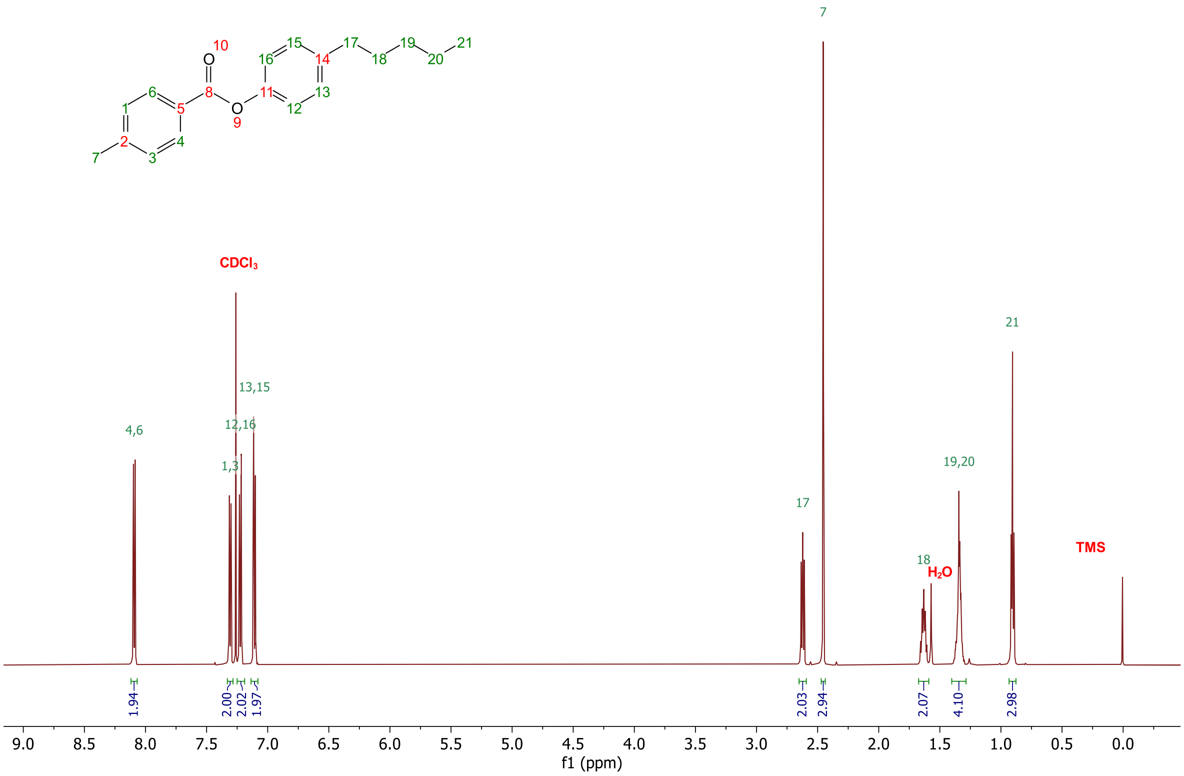

Figure S9. ^{1}H NMR spectrum of **CM**.

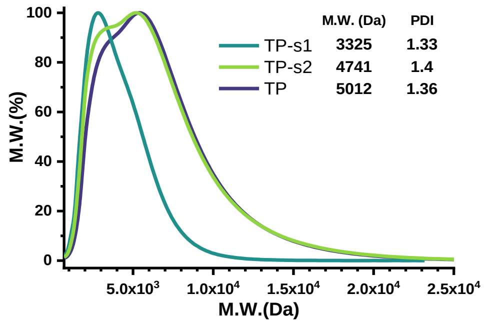

Figure S10. GPC molecular distributions of each synthesis step product of **TP**. Normalized by signal maximum.

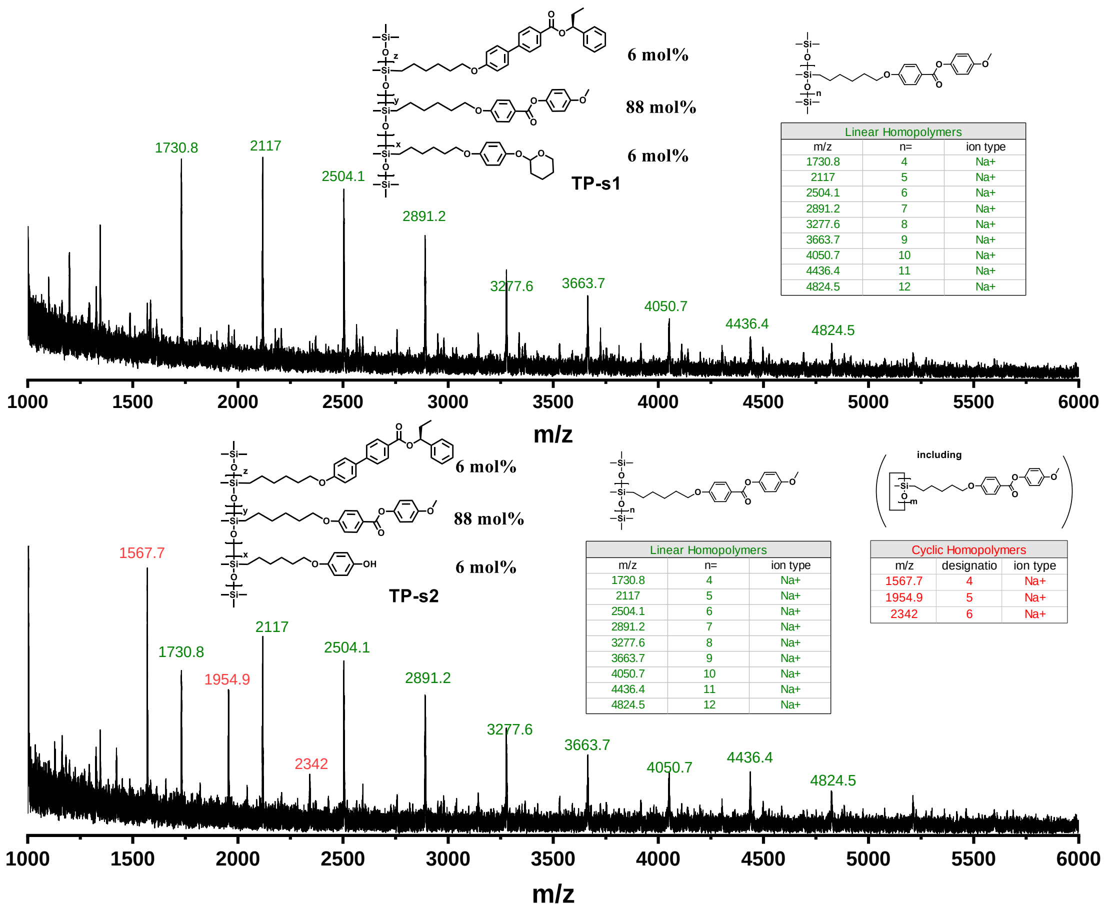

Figure S11. MALDI-TOF-MS spectra of **TP-s1**, **TP-s2**. Assignments of the **P1** homopolymer signals are specifically listed.

Table S1. Calculated rates of different chain lengths[1] (n) in **TP** that possess no acrylate group ($R_{0,n}$), one acrylate group ($R_{1,n}$), and more than two acrylate groups ($R_{2+,n}$). In mol%.

n=	$R_{0,n}$	$R_{1,n}$	$R_{2+,n}$
4	78.1%	19.9%	2.0%
5	73.4%	23.4%	3.2%
6	69.0%	26.4%	4.6%
7	64.8%	29.0%	6.2%
8	61.0%	31.1%	7.9%
9	57.3%	32.9%	9.8%
10	53.9%	34.4%	11.7%
11	50.6%	35.5%	13.9%
12	47.6%	36.5%	15.9%
13	44.7%	37.1%	18.2%
14	42.1%	37.6%	20.3%
15	39.5%	37.8%	22.7%
16	37.2%	37.9%	24.9%
17	34.9%	37.9%	27.2%
18	32.8%	37.7%	29.5%

[1] The actual **TP** has a wide distribution of chain lengths. Therefore, the general R_0, R_1 and R_{2+} of **TP** may be significantly influenced by such a complex, and can not be accurately specified. This table lists only the assumed most populated chain lengths of **TP**.

Formulas in the calculations:

$R_{0,n} = \boldsymbol{C}_n^0 \times 0.94^n$

$R_{1,n} = \boldsymbol{C}_n^1 \times 0.94^{n-1} \times 0.06$

$R_{2+,n} = 1 - R_{0,n} - R_{1,n}$

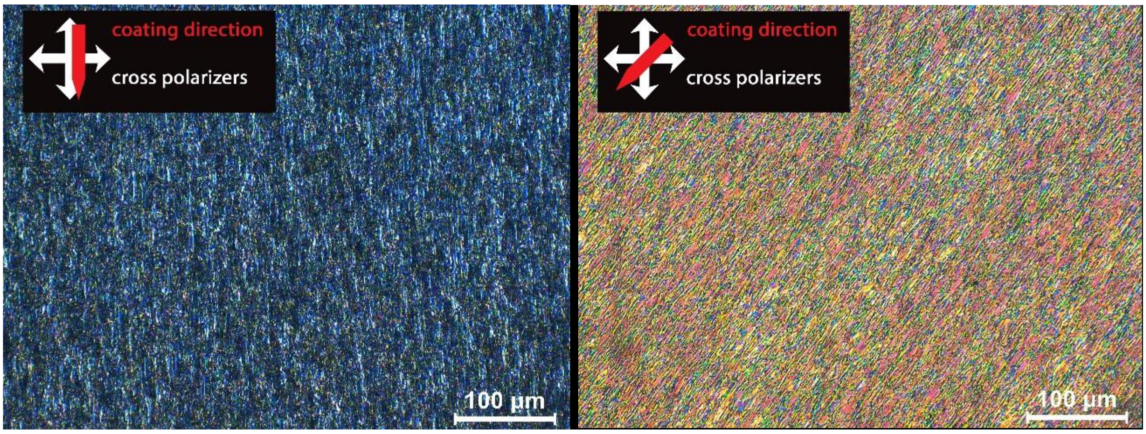

Figure S12. POM images of the bar-coated **TP** on a PI-glass substrate before curing. Images were taken with the transmission angles of the crossed polarizers set at 0°/90° and 45°/-45° to the shear direction.

Effect of photoinitiator concentration on the crosslinking completion

We investigated the cross linked fraction of the polymer coating as a function of photo initiator concentration (0.05% - 0.5%). These coatings were collected in 0.2 μm PTFE filters, and then filtrated by dichloromethane flushing. Proton NMR spectra were recorded of the eluents after evaporation of the solvent (Figure S13a). When 0.05 wt% of photoinitiator was used, which corresponds to ~1wt% compared to the total weight of the acrylic mesogens, it was found that acrylate groups were poorly converted, as the acrylate signals were still prominently present in the spectrum. When the photoinitiator concentration was increased, the peaks became less prominent, and at 0.25 wt% (5 wt% compared to the total weight of the acrylate groups), the acrylate signals were no longer observable, suggesting high conversion of the acrylate groups.

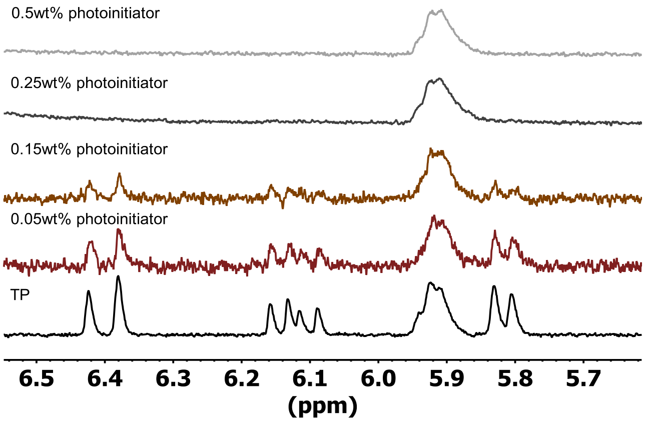

Figure S13. ^{1}H NMR spectra of the processed coatings from mixtures with different initiator concentrations, showing the zoomed-in region where the acrylates signals are present.

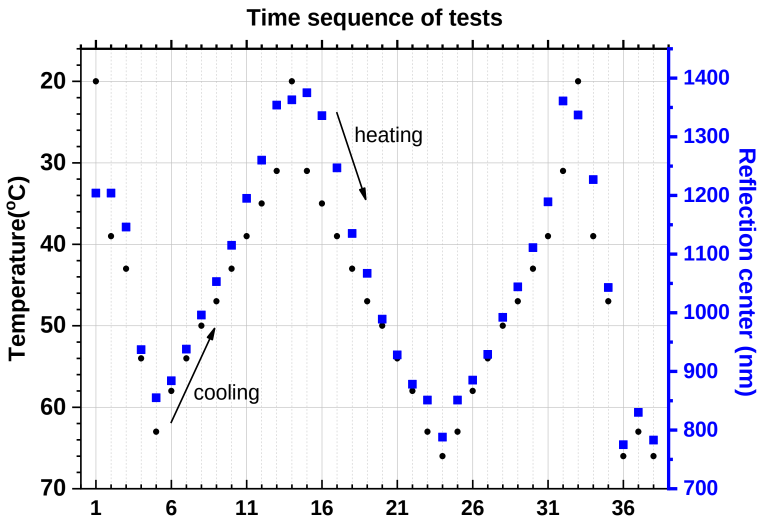

Figure S14. Diagram of the fully cured **TP** coating with reflection center wavelength against temperature during multiple heating and cooling rounds. Duration of each test is different, depending on the time needed for its optical properties to reach equilibrium at one temperature. The methodology of the temperature dependent spectra measurements is described in Materials and Methods session.

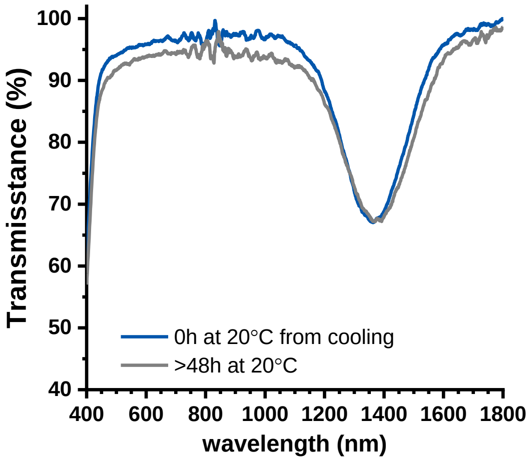

Figure S15. Transmission spectra of the fully cured **TP** coating at room temperature (20°C) right after cooling (Test#14 of Figure S14), and after 48 hours of storage at 20°C. The slight difference in transmittance is probably caused by variations at different measured spots.

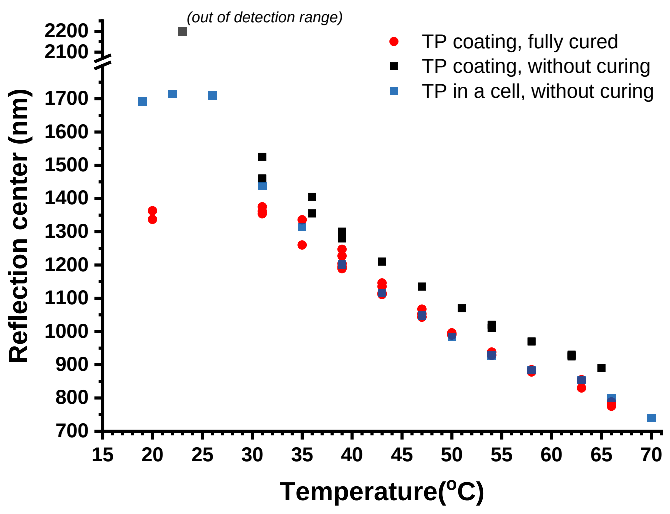

Figure S16. The reflection band center – temperature relation of the fully cured coating (using 0.5 wt% photoinitiator, red dots), compared to the coating by the same procedure but without the curing step (black squares) and the polymer in an alignment cell (blue squares). The reflection band of the **TP** coating without curing moved out of spectrometer detection range near room temperature.

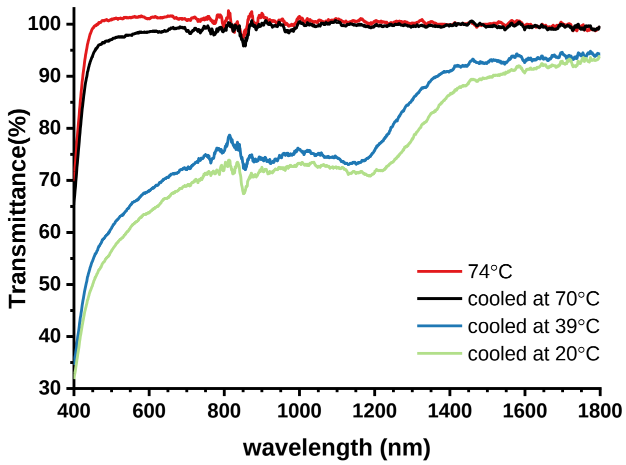

Figure S17. Transmission spectra of the fully cured **TP** coating taken during cooling from the isotropic temperature.

Kinetic behavior of the fully cured TP coatings.

We further analyzed the spectra to better understand the kinetic behavior of the temperature responsiveness. By heating the coating, the visible light transmittance dropped immediately, but slowly increased with time when maintaining the temperature (Figure S18a). Meanwhile, the reflection band center moved rapidly with temperature during heating (Figure S18b). These phenomena are due to scattering, which is caused by defects generation during mesogens rearrangement. The kinetics during cooling were different. Visible light transmittance remained high and unchanged with temperature (Figure S19a), but the reflection band center redshifted slowly (Figure S19b).

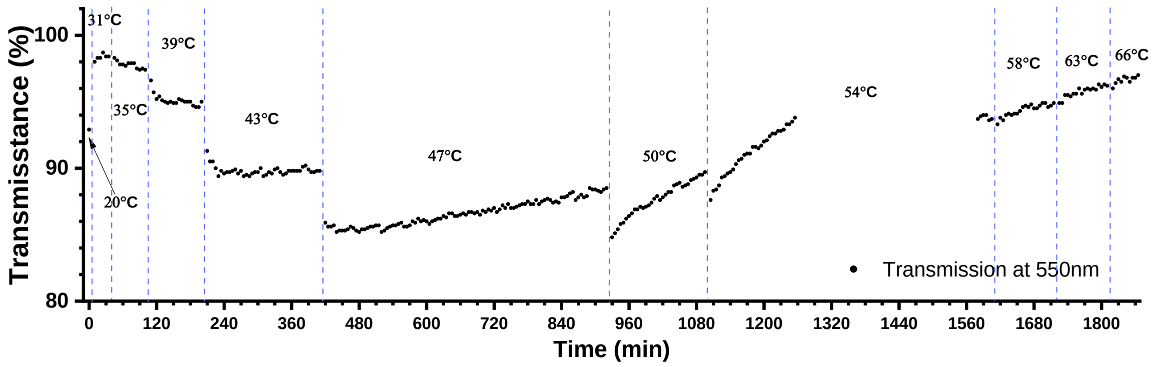

(a)

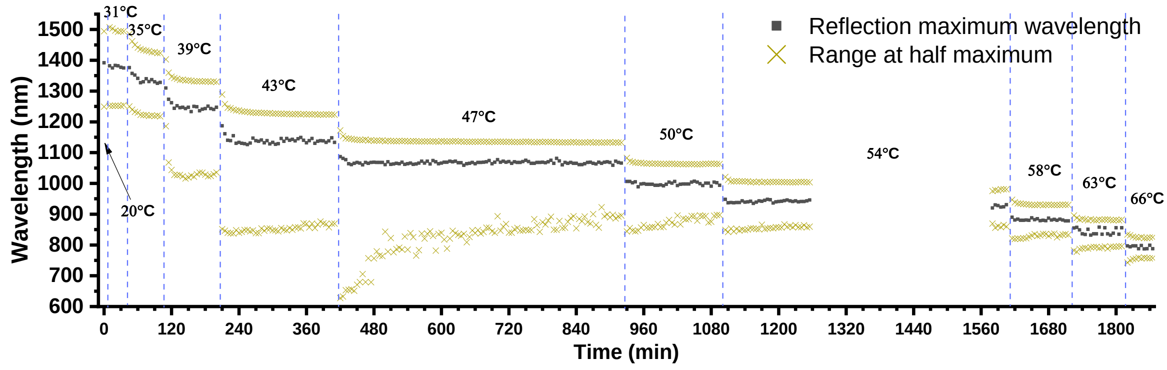

(b)

Figure S18. Kinetics of temperature response the cured **TP** photonic coatings during heating. (a) transmission at 550 nm representing scattering against time and (b) the position of the reflection band and reflection band width at half maximum against time.

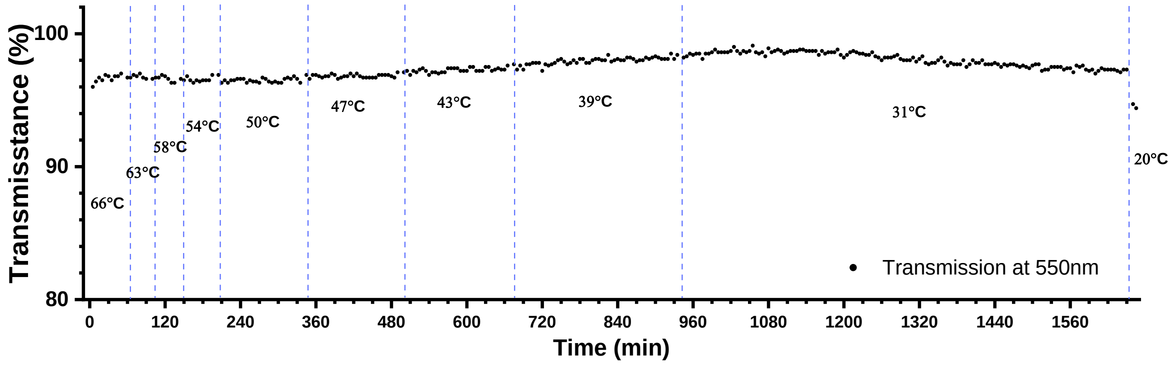

(a)

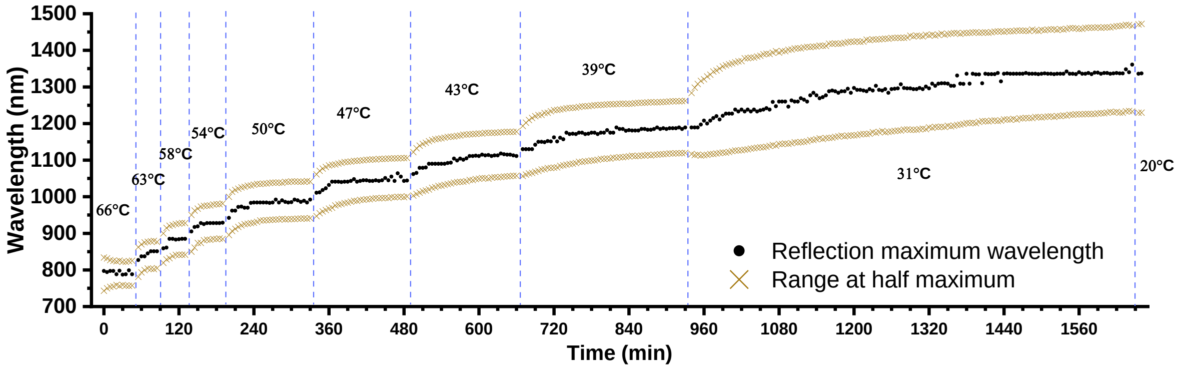

(b)

Figure S19. Kinetics of the fully cured **TP** coatings, showing: (a) transmission of 550 nm light against time and (b) Reflection band maximal wavelength and reflection band range at half maximum against time, during the cooling cycle of tests.

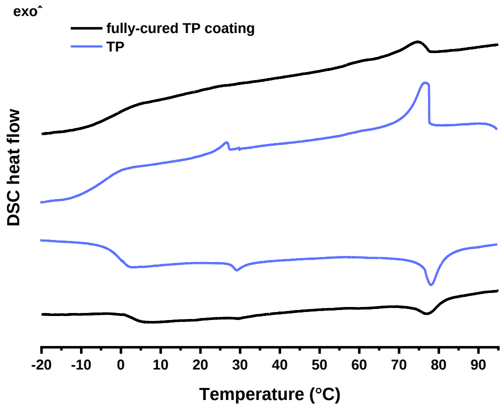

Figure S20. DSC curve of the **TP** coating before and after curing.

Polymer distribution of the completely polymerized TP coatings.

The crosslinked network fraction of the photonic coatings was estimated by filtration experiments. The coating was collected and weighted in a Teflon filter with a pore size of 0.2 μm and then filtrated with dichloromethane. The remaining solid in the filter was the insoluble part of the coating, and was therefore considered to be crosslinked polymers. 4 wt% of the original weight of the fully polymerized coating was left in the filter, while a control experiment of an uncured coating (made without using the photoinitiator and processed without the curing step) yielded 0 wt%. However, this 4 wt% does not represent all the reacted **TP**. The eluent from the filtration experiment contains a substantial amount of high M_w fraction as shown by GPC chromatography (Figure S21), with molecular weights as high as 200 kDa. We assume this fraction contains larger macromolecules consisting of multiple linked **TP** molecules that can pass the filter pores.

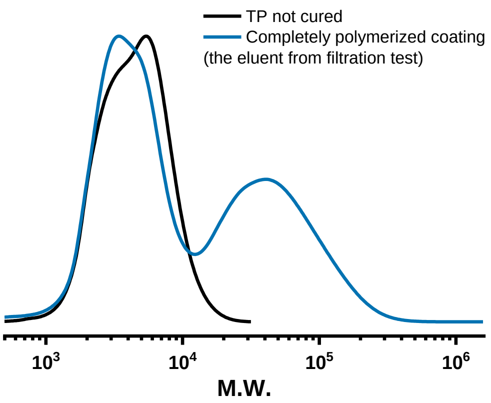

Figure S21. GPC spectra showing the molecular weight distributions of the completely polymerized **TP** coatings, compared to the original **TP**. Normalized by the intensity maximum of the signals.

References

S1. Wilderbeek, H.T.A.; Van Der Meer, M.G.M.; Jansen, M.A.G.; Nelissen, L.; Fischer, H.R.; Van Es, J.J.G.S.; Bastiaansen, C.W.M.; Lub, J.; Broer, D.J. Synthesis and properties of phenyl benzoate-based and biphenyl-based liquid crystalline thiol-ene monomers. *Liq. Cryst.* **2003**, *30*, 93–108.

S2. Kang, S.H.; Jang, K.S.; Theato, P.; Zentel, R.; Chang, J.Y. Photoimaging through in-situ photopolymerization of heterobifunctional mesogenic compounds in liquid crystalline state. *Macromolecules* **2007**, *40*, 8349–8354.

S3. Zhang, W.; Kragt, S.; Schenning, A.P.H.J.; De Haan, L.T.; Zhou, G. Easily Processable Temperature-Responsive Infrared-Reflective Polymer Coatings. *ACS Omega* **2017**, *2*, 3475–3482.

S4. Gray, G.W.; Hawthorne, W.D.; Lacey, D.; White, M.S.; Semlyen, J.A. 29Si N.M.R. investigations of polysiloxanes. *Liq. Cryst.* **1989**, *6*, 503–513.

S5. Engelhardt, G.; Jancke, H.; Mägi, M.; Pehk, T.; Lippmaa, E. Über die 1H-, 13C- und 29Si-NMR chemischen Verschiebungen einiger linearer, verzweigter und cyclischer Methylsiloxan-Verbindungen. *J. Organomet. Chem.* **1971**, *28*, 293–300.
